# The effect of quitting smoking on HDL-cholesterol - a review based on within-subject changes

**DOI:** 10.1186/2050-7771-1-26

**Published:** 2013-09-13

**Authors:** Barbara A Forey, John S Fry, Peter N Lee, Alison J Thornton, Katharine J Coombs

**Affiliations:** 1 P.N. Lee Statistics and Computing Ltd, Surrey, UK; 2 Independent consultant, Exeter, Devon, UK

**Keywords:** Smoking cessation, HDL cholesterol, Review, Meta-analysis

## Abstract

A higher concentration of high density lipoprotein cholesterol (HDL-C) in ex-smokers than smokers has consistently been observed. Better evidence of quitting effects comes from within-subject changes. We extend an earlier meta-analysis to quantify the reduction, and investigate variation by time quit and other factors. We conducted Medline and Cochrane searches for studies measuring HDL-C in subjects while still smoking and later having quit. Using unweighted and inverse-variance weighted regression analysis, we related changes (in mmol/l) to intra-measurement period, and estimated time quit, and to study type, location and start year, age, sex, product smoked, validation of quitting, baseline HDL-C, baseline and change in weight/BMI, and any study constraints on diet or exercise. Forty-five studies were identified (17 Europe, 16 North America, 11 Asia, 1 Australia). Thirteen were observational, giving changes over at least 12 months, with most involving >1000 subjects. Others were smoking cessation trials, 12 randomized and 20 non-randomized. These were often small (18 of <100 subjects) and short (14 of <10 weeks, the longest a year). Thirty studies provided results for only one time interval. From 94 estimates of HDL-C change, the unweighted mean was 0.107 (95% CI 0.085-0.128). The weighted mean 0.060 (0.044 to 0.075) was lower, due to smaller estimates in longer term studies. Weighted means varied by time quit (0.083, 0.112, 0.111, 0.072, 0.058 and 0.040 for <3, 3 to <6, 6 to <13, 13 to <27, 27 to <52 and 52+ weeks, p=0.006). After adjustment for time quit, estimates varied by study constraint on diet/exercise (p=0.003), being higher in studies requiring subjects to maintain their pre-quitting habits, but no other clear differences were seen, with significant (p<0.05) increases following quitting being evident in all subgroups studied, except where data were very limited. For both continuing and never smokers, the data are (except for two large studies atypically showing significant HDL-C declines in both groups, and a smaller decline in quitters) consistent with no change, and contrast markedly with the data for quitters. We conclude that quitting smoking increases HDL-C, and that this increase occurs rapidly after quitting, with no clear pattern of change thereafter.

## Introduction

It has long been known [1,2] that current smokers have a lower concentration of high density lipoprotein cholesterol (HDL-C) than do non-smokers. Evidence that ex-smokers have higher HDL-C concentrations than do current smokers (e.g. [3]) also exists, though partly based on between-group comparisons, with current smokers and quitters differing on other relevant variables. A method of investigating how quitting affects HDL-C, which better avoids confounding, uses within-subject estimates of change. In 2003, Maeda *et al*. [4] reviewed evidence of effects of quitting on lipid/ lipoprotein profiles, using data on within-subject changes. Their meta-analysis for HDL-C, based on 29 studies with follow-up periods from 30 days to 2 years or more, showed an increase following quitting of 0.100 (95% CI 0.074 to 0.127) mmol/l. The estimates were highly heterogeneous (p<0.001), though significant (p<0.05) variations were only seen by gender (greater increases in females) and by baseline HDL-C (greater increases for higher pre-quitting levels). No significant variation was seen by study design, publication year or intra-measurement period (increases similar for periods less or greater than 8 weeks).

Although more recent reviews have considered effects of quitting on HDL-C (e.g. [5-7]), none involve an updated meta-analysis. This review presents an updated meta-analysis using data on changes, more fully investigating how the increase following quitting varies by other factors. Of particular interest is quantifying how the increase varies with time, some authors claiming “a complete and very fast reversibility of changes after smoking cessation” [8] and others an initial quite rapid “movement towards normalization of HDL-C” which “will continue to progress toward normal (non-smoking) levels as long as cessation continues” [6]. Some studies reporting changes in quitters also report changes in continuing and never smokers. We summarize these data.

## Review

### Methods

#### Selection of studies and literature searches

For selected publications we examined abstracts, and where necessary full text, to find studies satisfying these inclusion criteria: (1) relevant to HDL-C and quitting; (2) clinical trial, human experimental, or epidemiological study; (3) prospective or longitudinal study; (4) at least five subjects quitting; (5) not restricted to those with coronary artery disease or taking antihyperlipidaemic drugs; (6) smoking habits recorded and blood sample taken concurrently on two or more occasions; (7) results reported separately for quitters during the study; (8) results available relevant to the change in HDL-C following quitting; (9) quitting for at least a day; and (10) results relate to conventional cigarette smoking.

Papers cited by Maeda *et al.*[4] were considered, the Medline searches they conducted being updated to July 2012. These used the search terms “(smoking OR tobacco OR cigar) AND (tobacco use cessation OR tobacco use cessation products OR stop OR quit OR cessation OR abstinence OR exsmokers) AND (cholesterol OR triglycerides OR lipoprotein OR HDL)”. New papers were then sought from Collaborative Trials within the Cochrane Library using the same keywords, from reference lists in accepted papers, and from an ongoing project on white blood cell changes (submitted for publication). The selected papers were then separated into studies, further study details being obtained from additional references if required.

#### Data entry

Relevant data were entered onto a study and a change database, each study being identified by a 6-letter reference (REF). The study database contains one record per study describing study attributes. The change database contains one or more records per study, describing estimates of HDL-C change from baseline.

Study attributes recorded include relevant publications, sexes considered, age range, location, years of start, finish and publication, length of follow-up, study design, nature of population studied (including smoking and medical criteria), study size, HDL-C measurement method, fasting or smoking abstinence requirements before measurement, diet or exercise modification during follow-up, and confounding and stratifying variables considered.

Details on the change database include smoking status (quitter, continuing smoker, never smoker), smoking habits at baseline and follow-up (products, cigarette types, amount smoked), biochemical validation methods, intra-measurement period, original measurement unit, data source, and population the data applies to (sex, age, intervention groups). Also recorded is information on the HDL-C change itself (mean change, or mean level at both baseline and follow-up), its variability (confidence limits, SD, SE, N, significance of change) and whether the change estimate was direct, or relative to never smokers or to continuing smokers. Where the mean change was unavailable, the median change, change in median, or an estimate based on the mean % change was accepted. Change data were entered for never smokers, continuing smokers and quitters, but not for smokers who quit during the follow-up period but resumed before the second HDL-C measurement. Change data were converted to mmol/l if necessary. Sex-specific data were preferred to combined-sex data. Data stratified on other variables were entered in addition to overall data, including stratification by later resumption of smoking, where available from studies with multiple follow-ups. Available information on baseline weight, body mass index (BMI) and other commonly reported physiological parameters, and on change in weight and BMI, was also recorded.

#### Statistical analysis

For trials giving results for subjects continuously abstinent since baseline, the quit time was taken as the intra-measurement period. For other trials, it was estimated from the study design details. For observational studies, where quitting could have occurred any time in an interval, the quit time was estimated based on the interval midpoint.

Non-stratified data were generally selected for analysis if available, with certain exceptions. Sex-specific data were preferred to combined-sex data. Where a study stratified the participants by time successfully quit, with data for more time points for persistent quitters than for those resuming smoking by the end of the study, the stratified data were used. Stratified data were also used where the levels were relevant to a factor considered in the heterogeneity analyses (see below). For many studies, the only estimates available were direct (not relative to changes in continuing or never smokers) and unadjusted for covariates. Therefore, to make estimates as comparable as possible between studies, direct estimates and those least adjusted for covariates were selected from studies providing a choice of estimates.

If missing for intermediate time points, changes in weight and BMI were estimated assuming linearity. Tertiles of baseline parameters and of change in weight and in BMI were derived from the combined data on quitters, continuing smokers and never smokers. The weight and BMI tertile variables were then merged, preferring the weight tertile when both were available.

For changes in HDL-C, unweighted and inverse-variance weighted means and SEs were estimated using repeated measures analysis of variance, accounting for serial correlations between changes at different times within the same group of subjects by the Kenward-Roger method [9,10]. For weighted analyses, an SE estimate was required for each change. If not provided, it was calculated from the p-value, 95% confidence interval (CI), or N and SD combined. Where information on variability was unavailable, the SE was estimated using the mean SD for changes where the SD was available.

The analyses of variance were conducted separately for quitters, continuing smokers and never smokers. Heterogeneity was investigated by intra-measurement period, estimated time quit, biochemical validation of quitting, study characteristics (type, continent, timing, constraint on diet/exercise), population details (age, sex), baseline levels of physiological parameters (HDL-C, LDL-C, triglycerides, systolic and diastolic blood pressure), and aspects of smoking before quitting. Change in weight was also included as a factor because it was considered important by several study authors, although interpretation is difficult as, unlike the other factors, this relates to changes concurrent with the HDL-C changes.

Formal within-study analysis of variation in change by intra-measurement period or by time quit for studies providing multiple estimates was not conducted, but such results if given by study authors are summarized.

To further assess effects of confounding, we compare change estimates from the same study that are comparable apart from level of adjustment. For direct estimates in quitters, the only possible factors are those relating to changes following quitting, such as BMI. Estimates relative to continuing or never smokers (or derived from a model involving two groups) may involve other factors.

#### Software

ROELEE Version 3.1 (P.N.Lee Statistics and Computing Ltd., Sutton, Surrey, UK) was used for data entry and some statistical analyses, and SAS Version 9.2 (SAS Institute Inc., Cary, North Carolina, USA) to conduct the repeated measures analysis of variance.

### Results

#### Literature searches

Table 1 summarizes the literature searches. 661 papers were considered, 27 cited by Maeda *et al.*[4], 615 identified from the July 2012 Medline search, 13 from the August 2012 Cochrane search, and six from other sources. 508 were rejected by examining abstracts, and 100 by examining full text. This left 53 papers, 47 providing primary results, and 6 reviews. The 47 primary papers described 45 distinct studies, one study (GEPNER) being described in three papers. Ten further papers provided background information.

**Table 1:** **Literature searches**^**a**^

PAPERS CITED BY PREVIOUS REVIEW [4]	
Publications examined 27	→ Rejects (reason 1) 2
↓	
Publications accepted (primary results) **25**^b^	
MEDLINE SEARCH 6^TH^ July 2012	
New abstracts examined 615	→ Rejects - total 500
↓	Reason 1 – 442; Reason 3 – 40; Reason 5 – 7; Reason 6 – 1; Reason 7 – 7; Reason 8 – 3;
Publications examined 115^c^	→ Rejects – total 91
↓	Reason 1 – 80; Reason 3 – 2; Reason 5 – 1; Reason 6 – 1; Reason 7 – 5; Reason 8 – 2
Publications accepted – total 24^c^	
Primary results **19**^c^	
Reviews 5	
Cochrane search 6^th^ August 2012	
New abstracts examined 13	→ Rejects – total 7
↓	Reason 1 – 5; Reason 8 – 2
New publications examined 6^d^	→ Rejects (reason 1) 6
SECONDARY REFERENCES 15^th^ August 2012^e^	
New abstracts examined 2	→ Rejects (reason 1) 1
↓	
New publications examined 1	→ Rejects (reason 1) 1
INFORMAL SEARCHING (including WBC review)	
New publications examined 4	
↓	
New publications accepted – total 4	
Primary results **3**	
Reviews 1	
**Total primary publications accepted – 47**	

^a^Reasons for rejection correspond to study inclusion/exclusion criteria (see Methods).

^b^Includes one paper [11] where HDL-C data not used in the earlier review [4].

^c^Includes one paper [12] initially rejected on the basis of the abstract but found to be relevant when examined for a separate review of WBC.

^d^One paper could not be adequately identified, but another paper [13] by the same author and apparently reporting the same study was substituted.

^e^Checking continued until no new publications were identified.

#### Studies

Table 2 summarizes details of the 45 studies, with further detail given in Additional file 1. Countries contributing the most studies were USA (15) and Japan (7). Eleven studies were randomized clinical trials (RCTs) of various smoking cessation treatments, while MOFFA3 was a RCT with an additional community control group. Twenty were non-randomized smoking cessation studies. These 32 trials were often small, 18 involving less than 100 subjects, and the largest (ALLEN and GEPNER) some 900. They were often short, 14 presenting results for less than 10 weeks, and the longest a year. The remaining studies were observational, three based around community health education studies, and ten on routine health checks or prospective studies. These were larger, most involving some thousands of subjects, and longer, all presenting changes over at least a year, and some for variable time periods. In particular, study BURNET recruited pre-menopausal women who returned for follow-up examination 1 and 2 years after reaching menopause.

**Table 2:** Study details

**Study REF**	**Source papers**	**Location**	**Study type**^**a**^	**Study duration**	**N**^**b**^	**Sex**^**c**^	**Baseline year**^**d**^
ALLEN	[14,15]	USA	RCT	6 weeks	935	B	1990
BASLER	[16]	Germany	RCT	3 months	139	B	1990
BURNET	[17,18]	USA	Obs	13 years	417	F	1983-84
ELIAS1	[19]	Sweden	Cess	8 weeks	40	M	1995
ELIAS2	[20]	Sweden	Cess	16 weeks	58	B	1995-96
FEHER	[21,22]	UK	Cess	2 weeks^e^	30	B	1988
FERRAR	[23]	USA	Cess	4 weeks	10	F	1999
FORTMA	[24,25]	USA	Obs/Int	3 years	40^f^	B	1979-80
GEPNER	[26-29]	USA	RCT	3 years	923	B	2005-07
GERACE	[30,31]	USA	Obs/Int	72 months	3470	M	1973-76
GREEN	[32]	Israel	Obs	1-4 years	968	M	1985-87
HAUSTE	[33-35]	Germany	Cess	6 months	197	M	2001
IINO	[36]	Japan	Cess	12 months	41	B	2002-03
KONDO	[12]	Japan	Cess	4 weeks	29	M	2003
KORHON	[37]	USA	RCT	15 weeks	130	F	2004-07
KUME	[38]	Japan	Obs	3 years	3053	B	2003-07
KUSHIM	[39]	Japan	Obs	5 years	1431	M	1985
LEE	[40]	Korea	Cess	2 months	20	M	2009
LUDVIK	[41,42]	Iceland	RCT	3 months	157	B	1989
MASARE	[43]	Australia	Cess	6 months	64	B	1984
MOFFA1	[44]	USA	Cess	60 days	36	F	1986
MOFFA2	[11]	USA	Cess	30 days	45	B	1991
MOFFA3	[45]	USA	RCT/Comm	77 days	43	B	1999
NIAURA	[46]	USA	RCT	12 weeks	28	F	1996
NILSSO	[47]	Sweden	RCT	4 months	400	B	1994
NORREG	[48]	Denmark	RCT	1 year	225	B	1994
PRIEME	[49]	Denmark	RCT	26 weeks	182	B	1996
PULS	[50]	Germany	Cess	5 weeks	218	B	2003-05
QUENSE	[51]	Sweden	Cess	2 weeks	24	M	1987
RABKIN	[52]	Canada	RCT	2-3 months	140	B	1982
RAHILL	[53]	USA	Obs	30 years	2280^g^	M	1961-70
RICHAR	[54]	France	Cess	3-12 weeks	101	B	1995
SHENNA	[55]	UK	Obs	4 years	41^f^	M	1980-83
STAMFO	[56]	USA	Cess	48 days/1 year	24	F	1984
STUBBE	[57]	Sweden	Cess	6 weeks	21	M	1981
SUWAZO	[58]	Japan	Obs	15 years	7058	M	1991-2002
SWANK	[59]	USA	Cess	7 weeks	19	F	1990
TAMURA	[60]	Japan	Obs/Int	4 years	1102	M	1999-2000
TERRES	[61]	Germany	Cess	24 weeks	121	B	1990-92
TONSTA	[62]^h^	Norway	RCT	1 year	55	B	1999-2000
VANDEN	[63]	Netherlands	Cess	1 year	106	M	1997-98
YAMAMO	[64]	Japan	Obs	3 years	7321	M	1989
YEH	[65,66]	USA	Obs	5 years	10892	B	1987-89
YOON	[67]	Korea	Obs	12 years	2848	M	1995-2006
ZHANG	[68]	China	Cess	3-6 months	67	M	2009-10

^a^RCT = randomised controlled trial of a smoking cessation aid or method. RCT/Comm = RCT with community control group. Cess = other smoking cessation study. Obs/Int = observational study as follow-up to an intervention study (other than of smoking cessation). Obs = other observational study.

^b^Total number of subjects in the study.

^c^B = both sexes, F = females, M = males.

^d^If not stated the year before publication is assumed.

^e^Subjects were studied 2 weeks before and 2 weeks after stopping smoking.

^f^Total number not stated, number shown is quitters FORTMA, SHENNA.

^g^Of the 2280 total participants, 1420 had at least 2 HDL-C measurements. An “observation” was taken as a successive pair of HDL-C measurements. Each person contributed between 1 and 9 (mean 4) observations to the total of 5895 observations analysed RAHILL.

^h^Results for a second study also reported by this paper were rejected as the subjects all had documented cardiovascular disease TONSTA.

Two studies involve persons with CHD risk factors and one diabetic patients. Seven are studies in people of low physical activity, and one in participants described as obese. One study excludes people with low BMI, while four exclude those with high BMI. Otherwise, populations seemed reasonably representative, though some studies were restricted to heavy or more addicted smokers, or excluded subjects with defined medical conditions (Additional file 1). Two studies started in the 1970s, 15 in the 1980s (including RAHILL which started recruitment in 1961 but only measured HDL-C from 1981 onwards), 19 in the 1990s and 9 in the 2000s. Descriptions of methods for blood sampling and HDL-C determination varied considerably, being summarized in Additional file 1, as are requirements for overnight fasting and/or smoking abstinence before measurement, though not all publications provided such details.

#### Changes in HDL-C following quitting

The available data on changes in HDL-C (mmol/l) following quitting are shown in Additional file 2. Two studies provided no change estimate, merely stating non-significance at p < 0.05. Study YAMAMO analysed HDL-C as a dichotomous variable, and reported that significantly more quitters than continuing smokers increased their HDL-C level from below to above 40 mg/dl. The 94 change estimates for the remaining 42 studies provide the main data set for analysis. They relate to quit times from 1 day to 157 weeks. For 23 estimates, the SE of the change was either given or could be derived, while for 71 it was estimated from N and an SD estimate (0.263) derived from other studies.

The data concern 53 independent sets of subjects, including three from TAMURA, an observational study presenting results stratified by time between quitting and follow-up. Four studies provide sets stratified by sex, three by persistent quitting vs. later resumption, and one each by exercise training vs. control and by weight gain. The remaining 32 studies provide one set each. In addition to TAMURA, thirteen studies provide data on changes in non-independent multiple periods. The most intensive study was MOFFA2 presenting results for 11 periods up to 30 days, while four periods were presented by HAUSTE (up to 26 weeks) and by STUBBE and VANDEN (up to 52 weeks). Some additional data (not included in the analysis) are shown in footnotes.

**Figure 1:**
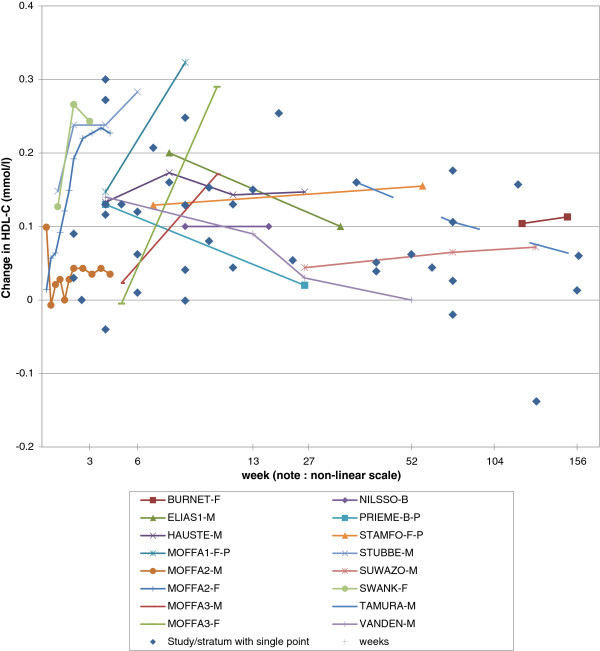
**Change in HDL-C by time since quitting.** The data are also shown in Additional file 2 (Table A2-1). Independent estimates for different times from study TAMURA are joined by a dashed line. Non-independent estimates for different times from other studies are joined by solid lines and distinguished by symbol and colour, as shown in the figure legend. Where there is a single estimate for a study/stratum, this is shown as a blue diamond: Week 2 FEHER-B, QUENSE-M, PULS-B. Week 4 FERRAR-F, KONDO-M, MOFFA1-F-R, NORREG-B, PRIEME-B-R. Week 5 RICHAR-B. Week 6 ALLEN-B, MASARE-M, MASARE-F. Week 7 STAMFO-F-R. Week 8 ELIAS2-B. Week 9 BASLER-B, LEE-M, NIAURA-F-E, NIAURA-F-C. Week 10 RABKIN-M, RABKIN-F. Week 12, KORHON-F, TONSTA-B. Week 13 LUDVIK-B. Week 19 ZHANG-M. Week 23 TERRES-B. Week 44 YOON-M-L, YOON-M-H. Week 52 GEPNER-B. Week 65 GREEN-M. Week 78, FORTMA-B, RAHILL-M, YEH-B. Week 119 SHENNA-M. Week 130 KUSHIM-M. Week 156 GERACE-M. Note that each individual independent point and the start of each group of non-independent points effectively joins back to the (0,0) point, but this is not shown in the Figure for clarity. Note also that the scale on the x-axis is non-linear (with distances between 13 and 52 weeks reduced to a quarter, and over 52 weeks to a tenth). Abbreviations: B = both sexes, F = females, M = males; L=low weight gain (<1.3 kg), H=high weight gain (≥1.3 kg); P = persistent quitter, R = resumed smoking before end of study; E = exercise training intervention, C = control group.

The data are also shown in Figure 1, the x-axis showing time since quitting on a non-linear scale. The independent points from TAMURA are joined by a dotted line, while points for studies contributing multiple non-independent data are joined by solid lines. For these studies the available information is insufficient to determine whether variations between successive follow-ups are statistically significant, despite about half using repeated measures methods to analyze their data.

As seen in Figure 1, several studies show a rapid increase in the first three weeks or so after quitting (FEHER, MOFFA2-females, STUBBE, SWANK), but little or no increase was seen in that time span from other studies (QUENSE, PULS), the exceptional pattern for MOFFA2-males showing an increase at day 2 subsequently dropping back to only a small increase from baseline. For quit times longer than 3 weeks, most data points show an increase in HDL-C, with only five decreases, of which only the largest from study KUSHIM (change −0.138) was significant (p<0.05). Of the longer-term studies with multiple quit times, some (HAUSTE, NILSSO, SUWAZO) show quite similar increases at the different times studied, one (MOFFA1) suggests an increasing change with time, another (MOFFA3, both sexes) suggests a change only starting after week 5, while others (ELIAS1, PRIEME, VANDEN) suggest a return towards baseline after an initial increase.

Table 3 summarizes the repeated measures analyses. The mean change was 0.107 (95% CI 0.085 to 0.128) unweighted and 0.060 (95% CI 0.044 to 0.075) weighted, the difference due to the absence of larger increases (>0.2) in the longer-term large observational studies (notably GERACE).

**Table 3:** **Estimates of change in total HDL-C (mmol/l) following quitting**^**a**^

**Factor**	**Level**	**N**^**b**^	**Unweighted analysis**		**Inverse-variance weighted analysis**	
			**Mean (95% CI)**	**p**^**c**^	**Mean (95% CI)**	**p**^**c**^
Overall		94	0.107 (0.085 to 0.128)	<0.001	0.060 (0.044 to 0.075)	<0.001
**Univariate models**						
Period^d^	<3 weeks	21	0.102 (0.047 to 0.157)	0.04	0.100 (0.032 to 0.169)	<0.001
	3 to <6	21	0.102 (0.063 to 0.141)		0.082 (0.042 to 0.123)	
	6 to <13	20	0.157 (0.119 to 0.194)		0.131 (0.094 to 0.168)	
	13 to <27	11	0.107 (0.057 to 0.158)		0.108 (0.061 to 0.155)	
	27 to <52	1	0.100 (−0.068 to 0.268)		0.094 (−0.221 to 0.409)	
	52+ weeks	20	0.065 (0.027 to 0.102)		0.041 (0.026 to 0.057)	
Time quit^e^	<3 weeks	23	0.101 (0.050 to 0.152)	NS	0.083 (0.030 to 0.137)	0.006
	3 to <6	20	0.115 (0.075 to 0.155)		0.112 (0.069 to 0.154)	
	6 to <13	22	0.145 (0.109 to 0.181)		0.111 (0.075 to 0.148)	
	13 to <27	9	0.099 (0.042 to 0.156)		0.072 (0.036 to 0.109)	
	27 to <52	4	0.088 (0.003 to 0.173)		0.058 (−0.004 to 0.121)	
	52+ weeks	16	0.062 (0.020 to 0.105)		0.040 (0.023 to 0.057)	
**Models adjusted for time quit**						
Max Age^f^	<50 years	8	0.124 (0.047 to 0.201)	NS	0.161 (0.072 to 0.250)	NS
	50-70 years	28	0.107 (0.071 to 0.144)		0.065 (0.038 to 0.093)	
	>70 years	58	0.094 (0.063 to 0.125)		0.080 (0.057 to 0.104)	
Sex	Male	46	0.090 (0.060 to 0.121)	NS	0.073 (0.046 to 0.099)	NS
	Female	30	0.139 (0.094 to 0.184)		0.123 (0.061 to 0.186)	
	Combined	18	0.095 (0.052 to 0.138)		0.078 (0.050 to 0.107)	
Continent	N America	49	0.121 (0.082 to 0.160)	NS	0.055 (0.024 to 0.087)	NS
	Europe	30	0.110 (0.075 to 0.146)		0.096 (0.062 to 0.130)	
	Asia	13	0.067 (0.016 to 0.118)		0.081 (0.050 to 0.112)	
	Australia	2	0.026 (−0.099 to 0.152)		0.018 (−0.179 to 0.215)	
Timing^g^	<1990	26	0.119 (0.077 to 0.160)	NS	0.049 (0.016 to 0.082)	0.08
	1990-1999	58	0.100 (0.071 to 0.129)		0.088 (0.065 to 0.110)	
	2000+	10	0.071 (0.012 to 0.130)		0.077 (0.040 to 0.114)	
Study type	Observational	16	0.094 (0.031 to 0.157)	NS	0.086 (0.057 to 0.115)	NS
	RCT	16	0.093 (0.042 to 0.145)		0.090 (0.057 to 0.123)	
	Other	62	0.108 (0.073 to 0.143)		0.067 (0.040 to 0.093)	
Product smoked	Any	16	0.102 (0.056 to 0.148)	NS	0.103 (0.072 to 0.133)	0.09
	Cigarettes	67	0.102 (0.075 to 0.130)		0.069 (0.047 to 0.091)	
	Cigarettes only	11	0.095 (0.035 to 0.155)		0.092 (0.054 to 0.129)	
Validation of quitting	Yes	48	0.113 (0.077 to 0.149)	NS	0.088 (0.060 to 0.115)	NS
	No	46	0.092 (0.059 to 0.125)		0.075 (0.051 to 0.098)	
Constraint on diet/exercise	Stay same	15	0.141 (0.089 to 0.193)	0.09	0.191 (0.126 to 0.255)	0.003
	Improve	9	0.135 (0.076 to 0.193)		0.064 (0.036 to 0.092)	
	No constraint	70	0.087 (0.061 to 0.114)		0.082 (0.066 to 0.099)	
Baseline HDL-C	1st tertile	34	0.111 (0.070 to 0.151)	NS	0.101 (0.068 to 0.134)	0.03
	2nd tertile	25	0.131 (0.083 to 0.178)		0.097 (0.048 to 0.147)	
	3rd tertile	28	0.100 (0.064 to 0.135)		0.084 (0.061 to 0.108)	
	unknown	7	0.044 (−0.030 to 0.118)		0.042 (0.010 to 0.073)	
Baseline weight/BMI^h^	1st tertile	19	0.132 (0.066 to 0.199)	NS	0.154 (0.042 to 0.267)	NS
	2nd tertile	28	0.073 (0.039 to 0.108)		0.073 (0.048 to 0.098)	
	3rd tertile	21	0.111 (0.060 to 0.163)		0.112 (0.065 to 0.158)	
	unknown	26	0.120 (0.083 to 0.156)		0.076 (0.047 to 0.105)	
Change in weight/BMI^h^	1st tertile	5	0.155 (0.077 to 0.232)	NS	0.161 (0.092 to 0.230)	0.06
	2nd tertile	29	0.076 (0.031 to 0.121)		0.074 (0.022 to 0.125)	
	3rd tertile	51	0.100 (0.073 to 0.128)		0.065 (0.041 to 0.089)	
	unknown	9	0.105 (0.041 to 0.170)		0.104 (0.042 to 0.166)	
Baseline LDL-C	1st tertile	17	0.067 (0.001 to 0.132)	NS	0.092 (0.045 to 0.139)	NS
	2nd tertile	16	0.132 (0.063 to 0.201)		0.084 (0.017 to 0.150)	
	3rd tertile	17	0.085 (0.039 to 0.130)		0.071 (0.028 to 0.113)	
	unknown	44	0.110 (0.079 to 0.141)		0.080 (0.054 to 0.106)	
Baseline Triglycerides	1st tertile	26	0.134 (0.085 to 0.184)	NS	0.099 (0.017 to 0.181)	NS
	2nd tertile	13	0.108 (0.059 to 0.157)		0.099 (0.034 to 0.163)	
	3rd tertile	24	0.075 (0.027 to 0.123)		0.079 (0.044 to 0.114)	
	unknown	31	0.096 (0.062 to 0.130)		0.075 (0.050 to 0.099)	
Baseline Systolic BP	1st tertile	8	0.079 (0.015 to 0.142)	NS	0.060 (0.006 to 0.115)	NS
	2nd tertile	7	0.117 (0.046 to 0.188)		0.096 (0.058 to 0.134)	
	3rd tertile	5	0.066 (−0.016 to 0.148)		0.090 (0.056 to 0.125)	
	unknown	74	0.107 (0.079 to 0.135)		0.075 (0.052 to 0.098)	
Baseline Diastolic BP	1st tertile	8	0.079 (0.015 to 0.142)	NS	0.060 (0.006 to 0.115)	NS
	2nd tertile	7	0.117 (0.046 to 0.188)		0.096 (0.058 to 0.134)	
	3rd tertile	5	0.066 (−0.016 to 0.148)		0.090 (0.056 to 0.125)	
	unknown	74	0.107 (0.079 to 0.135)		0.075 (0.052 to 0.098)	

^a^See Additional file 2 (Table A2-1) for data used on change and SE. Analyses are based on repeated measures analysis of variance using the Kenward-Roger method.

^b^Number of estimates that the mean is based on.

^c^NS p≥0.1. For the overall analysis, this is based on a test for an increase following quitting. For the subgroup analysis, this tests for variation by subgroup level.

^d^Period between first measurement when smoking and second measurement when not smoking (using mean, median or midpoint where necessary).

^e^Best estimate of time since quit at second measurement.

^f^Maximum age of population at baseline.

^g^Based on year of start of study.

^h^Using data for weight if both weight and BMI available (see Methods).

In both unweighted and weighted analysis, there was significant (p<0.05) variation by intra-measurement period. For time quit, the variation was significant only in the weighted analysis, although the pattern of a lower, but still significant, increase in the last interval (52+ weeks) remained evident. When tested in univariate analyses (Additional file 3), several other factors showed significant variation, particularly in the weighted analysis (age, sex, continent, timing, constraint on diet/exercise, baseline HDL-C, change in weight/BMI). However many of these factors are inter-correlated, and correlated with time quit (e.g. observational studies had longer times and less validation of quitting or constraint on diet/exercise). In the further analyses in Table 3, we included time quit in the model (chosen as perhaps more biologically relevant than intra-measurement period). Except where based on few change estimates, increases following quitting were significant at each level of each factor. Inclusion of time quit markedly reduced the number of other factors showing significant variation between levels, with none significant in the unweighted analysis. In the weighted analysis, HDL-C increases were greater (p=0.003) in studies requiring subjects to maintain their pre-quitting diet and exercise habits than in studies encouraging healthier diets or more exercise, or imposing no constraint. Variation by baseline HDL-C was significant (p=0.03), but due to smaller changes in the studies where baseline HDL-C was unavailable, rather than to variation between the tertiles. There was also some evidence (p=0.06), that increases were greater in those with least weight gain following quitting.

Although no study presented data by amount smoked or any other aspect of pre-quitting smoking habits, a few provided data (see footnotes in Additional file 2) on HDL-C change by other factors, such as age and dose of nicotine patch. No significant (p<0.05) variation in change by any such factor was seen.

Several studies provided information on the effect of confounder adjustment (Table 4). In some, comparison is only possible between an unadjusted or age-adjusted estimate and a multiply-adjusted estimate, so inferences cannot be drawn concerning effects of adjustment for specific variables. However in two studies (KUSHIM, SUWAZO) estimates are somewhat higher when adjusted for BMI change, in one (STUBBE), the estimate is lower with adjustment for dietary fat change, while in study PRIEME adjustment for baseline HDL-C had little effect on change relative to continuing smokers. The significance of these differences cannot be assessed.

**Table 4:** **Effects of adjustment on change in HDL-C (mmol/l) following quitting**^**a**^

**REF**	**Sex or strata**^**b**^	**Period**^**c**^	**Less adjusted**		**More adjusted**	
			**Factors**	**Change**	**Factors**	**Change**
GERACE^d^	M	313 (156)	none	0.021	age, baseline HDL-C, serum thiocyanate, weight change, Hegsted score change, medications, alcohol, DBP	0.062
GREEN^e^	M	52-209 av 130 (65)	age	0.044	+ change in BMI, alcohol, coffee, sport	0.044
KUSHIM	M	261 (130.5)	none	−0.138	change in BMI	−0.092
PRIEME^d^	B	4	none	0.122	baseline HDL-C	0.120
RAHILL^d^	M	156 (78)^f^	age	0.026	+ baseline HDL-C, LDL-C, diabetes, hypertension, CVD, CHD, medications	0.041
STUBBE	M	2	none	0.238	change in dietary fat	0.179
SUWAZO^g^	M	52 (26)	age, day/shift work, alcohol, exercise	0.044	+ change in BMI	0.067
		104 (78)		0.065		0.100
		156 (130)		0.072		0.098
YOON	M^g^	52-156 av 88 (44)	None	0.045	age, amount smoked, time between measurements, education, economic status, marital status, diabetes, hypertension, alcohol, inactivity	0.045
	M-L^h^			0.051		0.047
	M-H^h^			0.039		0.031

^a^Data are shown for estimates of change in HDL-C which are equivalent except for the level of adjustment.

^b^B = both sexes, F = females, M = males; L=low weight gain (<1.3 kg), H=high weight gain (≥1.3 kg).

^c^Period (weeks) between first measurement when smoking and second measurement when quitting. The estimated time quit is shown in parentheses, if different. “av” = average.

^d^Change is relative to continuing smokers (change in quitters minus change in continuing smokers) GERACE, PRIEME, RAHILL.

^e^Change is relative to never smokers (change in quitters minus change in never smokers) GREEN.

^f^Results are adjusted to 3 year interval RAHILL.

^g^Adjusted change estimated from a two-group analysis (quitters and continuing smokers) SUWAZO, YOON.

^h^Adjusted change estimated from a two-group analysis (low and high weight gain) YOON.

#### Changes in continuing smokers and never smokers

Change data for continuing smokers and never smokers from 20 studies are also shown in Additional file 2. For both groups the data are reasonably consistent with no change, and contrast markedly with the quitter data. In most studies, changes are minor and not significant, although two large studies (KUSHIM, YEH), which also surprisingly reported decreases in quitters, reported large decreases for both never and continuing smokers. The overall estimates from the repeated measures analyses (Additional file 3) were not significant except for the weighted analysis of never smokers, which showed an overall decrease (change = −0.048, p=0.004), due to the contributions of KUSHIM, and particularly YEH.

### Discussion

Despite considerable between-study variation in design, methods and populations, and many studies involving few subjects, the data, with few exceptions, show that HDL-C increases following quitting. From repeated measures analysis, mean increases (mmol/l) were 0.107 (95% CI 0.085 to 0.128) unweighted and 0.060 (95% CI 0.044 to 0.075) inverse-variance weighted.

We present some analyses by the period between measurements taken when still smoking and when quit. However, quitting may not start at baseline, and we also present analyses by estimated time quit. To derive this, studies were divided into three types. For cessation trials with quitters required to be continuously abstinent, intra-measurement period and time quit are identical. For other trials, where quitting may not have started immediately, time quit can be derived from the study design details. For observational studies, where quitting could have started at any time in an interval, we estimate time quit from the interval midpoint. This may be questionable, but should not affect our conclusions.

An increase in HDL-C was evident in subgroups by sex, age, location, timing, study type, baseline HDL-C, baseline weight, increase in weight, or whether quitting was validated biochemically. Our observation that quitting is associated with increased HDL-C, based on within-subject changes, is consistent with considerable evidence of lower HDL-C levels in smokers than quitters [1,2]. A claim [6] that HDL-C in quitters increases with increasing time quit is not clearly confirmed by our analyses. Within the first year following quitting, the increase shows little overall variation by time quit, with different studies showing different patterns, and although estimates were lower for longer quit times, this difference is open to doubt. Notably, two large observational studies (KUSHIM, YEH) reported a decline in HDL-C following quitting, and an even larger decline in continuing smokers and never smokers, results inconsistent with other studies. Could some systematic difference between baseline and follow-up have biased the estimates of change from these studies, studies which contributed substantially to the lower estimates associated with longer-term quitting, and the lower weighted than unweighted overall estimate.

Previously, Maeda *et al*. [4], based on far fewer change estimates, found significantly (p<0.05) higher increases in HDL-C for females than males. Though our estimate was also higher for females, we did not find the difference to be significant. Nor could we repeat their finding that increases following quitting were significantly higher in those with higher levels before quitting, though we did find some trend in that direction. We studied some factors not considered in the earlier review [4]. The only significant result we found was that increases were substantially higher in trials where subjects were required to maintain pre-quitting diet and exercise habits (0.191 mmol/l) than in those that encouraged healthier diets or more exercise (0.064 mmol/l), or studies that did not impose any constraint (0.082 mmol/l). Taken together with the non-significant tendency for higher increases where subjects had less weight gain, and higher increases when adjusted for weight gain in two studies, the results suggest a role of diet and weight gain in mitigating the magnitude of the increase. We found no clear evidence that increases in HDL-C following quitting differed by mean age of subjects in the trial, study location, product smoked, validation of quitting, or baseline weight/BMI, all factors not considered earlier [4]. We also found no clear evidence that change estimates varied systematically by level of confounder adjustment.

Compared to the previously published meta-analysis [4] we consider many more studies, and studied effects of quitting by a much longer list of factors. However, the evidence considered must still be regarded as limited in a number of respects. Thus, many studies involved few subjects, no study provided data relating the change following quitting to initial amount smoked, no clinical trial investigated changes over more than a year, and few studies provided evidence on the effect of adjustment for confounders. Also, the variability of changes was often unavailable, and had to be estimated from other studies. Finally, many studies considered provided no evidence on changes in continuing smokers and in never smokers, though the available data are reasonably consistent with a lack of change, and contrast markedly with the results for quitters.

The mechanisms by which smoking might decrease or quitting might increase HDL-C are not fully understood. One popular explanation [6,69] is that smoking alters catecholamine release, and hence free fatty acid release, which in turn affects VLDL and LDL concentrations to favour their accumulation in blood, contributing to a lower HDL concentration. However other hypotheses have been proposed [4,70], involving smoking increasing cholesteryl ester transfer protein, reducing lecithin cholesterol acyltransferase activity, affecting apo-1 synthesis, or increasing triglycerides. It has also been suggested that some of the change in HDL-C following quitting is due to associated changes in diet [43,51,56,57], but as mentioned above our analyses indicated that the increase in HDL-C tended to be greater in those with least weight gain or dietary change.

### Conclusions

Quitting smoking is clearly associated with an increase in HDL-C concentrations. Generally the increase occurs rapidly, in less than three weeks, with no clear pattern of change thereafter. This emphasises that some, at least, of the adverse effects of smoking appear to be rapidly reversible on quitting, strengthening the argument for encouraging smokers to quit.

## Abbreviations

BMI: Body mass index; CI: Confidence interval; HDL-C: High density lipoprotein cholesterol; LDL-C: Low density lipoprotein cholesterol; N: Number of subjects; REF: 6-letter study reference code; RCT: Randomised clinical trial; SD: Standard deviation; SE: Standard error; VLDL: Very low density lipoprotein; WBC: White blood cells.

## Competing interests

PNL, founder of P.N. Lee Statistics and Computing Ltd., is an independent consultant in statistics and adviser in epidemiology and toxicology to various tobacco, pharmaceutical and chemical companies. JSF, BAF and KJC work for, and AJT consults for, P.N. Lee Statistics and Computing Ltd.

## Authors’ contributions

PNL and BAF planned the study, AJT carried out the literature search, KJC extracted and entered the data which were checked by BAF. JSF and BAF carried out the statistical analyses. The report was drafted by PNL and BAF, with the other authors commenting on it. All authors read and approved the final manuscript.

## Supplementary Material

Additional file 1 **Further study details.** This file presents details, additional to those shown in Table 2, of the design of each study and values of baseline characteristics of the study populations. Click here for file

Additional file 2 **Changes in HDL-C, bodyweight and BMI.** This file presents the data on change in HDL-C, body weight and BMI for each available intra-measurement period, separately for quitters, continuing smokers and never smokers. Click here for file

Additional file 3 **Further analyses.** This file presents results of analyses of HDL-C change in quitters additional to those shown in Table 3. It also shows results of equivalent analyses for continuing and never smokers. Click here for file
