# Supplementary material for: The effect of quitting smoking on HDL-cholesterol - a review based on within-subject changes
Source: Biomark Res. 2013 Sep 13;1:26. doi: 10.1186/2050-7771-1-26 (PMC4177613; doi:10.1186/2050-7771-1-26)
Supplement: Additional file 1 — Further study details. This file presents details, additional to those shown in Table 2, of the design of each study and values of baseline characteristics of the study populations. [file 2050-7771-1-26-S1.docx]

**The effect of quitting smoking on HDL-Cholesterol - A review based on within-subject changes**

# Barbara A Forey, John S Fry, Peter N Lee, Alison J Thornton and Katharine J Coombs

# Additional File 1 – Further study details

Table 2 in the main paper gives brief information about each study, including references to the source papers. Additional File 1 gives further detail.

Table A1-1 provides additional detail on study design, including study type and duration, information on relevant smoking cessation aids and methods for cessation trials, any smoking-related criteria for study participation, and other major inclusion criteria. It also shows the sexes and ages included in the study, and gives information on any requirements for fasting or smoking abstention before blood samples were taken for HDL-C determination.

Table A1-2 shows whether the study inclusion criteria involved any aspect of weight, diet, exercise or other traditional coronary risk factors, and whether the conduct of the study involved any constraint, control or modification of diet or exercise.

Table A1-3 gives further information on medical inclusion criteria, while Table A1-4 gives information on laboratory methods for determining HDL-C.

Table A1-5 gives baseline values, where available, for various physiological parameters.

Table A1-6 shows the product smoked, the mean amount smoked before quitting, and whether quitting was biochemically validated. For studies also providing HDL-C change data for continuing smokers, equivalent baseline smoking data are shown. Definitions of non-smoking groups are also shown.

## Table A1-1 Study description and design

| Study REF | Location | Brief study description^[[1]](#endnote-1)^ | Sex^[[2]](#endnote-2)^ | Age | Fasting^[[3]](#endnote-3)^ |
| --- | --- | --- | --- | --- | --- |
| ALLEN | USA, nationwide, 9 centres | 6 week RCT of patches for cessation in smokers of 20+/day for 1y, not using other tobacco, quit attempt in last y | B | 21-65 | overnight^[[4]](#endnote-4)^ |
| BASLER | Germany, Hanover | 3 month RCT of gum for smoking cessation in smokers with | B | Any |  |
| BURNET | USA, Allegheny County, Pa. | 13 year study of women reaching menopause, with follow-up 1 and 2 years after menopause, licensed drivers | F | 42-50 | overnight |
| ELIAS1 | Sweden, Goteborg | 8 week cessation study in smokers of 10+/day for 20y | M | 40-60 | overnight^[[5]](#endnote-5)^ |
| ELIAS2 | Sweden, Goteborg | 8 week reduction then 8 week cessation study with nasal spray in smokers of 15+/day for 3y not using smokeless tobacco | B | 18+ |  |
| FEHER | UK, London | 2 week cessation study in smokers of 7+/day for 5y^[[6]](#endnote-6)^ | B | 20-68 |  |
| FERRAR | USA, Baltimore Md., Washington DC | 4 week cessation study | F | Any (premenopausal) | overnight^e^ |
| FORTMA | USA, Cal. | 3 year follow-up study of community health education intervention^[[7]](#endnote-7)^ | B | 25-74 |  |
| GEPNER | USA, Madison, Milwaukee Wis. | 3 year RCT of 5 smoking cessation pharmacotherapies in smokers of 10+/day for 6m | B | 18+ | yes (unspecified) |
| GERACE | USA, nationwide, 22 centres | 72 month follow-up of intervention program^[[8]](#endnote-8)^ | M | 35-57 | overnight |
| GREEN | Israel | Factory worker study with follow-up after 1-4 years | M | 20-64 | overnight |
| HAUSTE | Germany, Erfurt | 6 month smoking cessation study of patches and gum in smokers of >20/day for 5y and Fagerstrom score ≥5 | M | 25-45 |  |
| IINO | Japan, Fukuoka | 12 month cessation study of patches in smokers of 5+/day for 2 y | B | Any | overnight |
| KONDO | Japan, Nagoya | 4 week cessation study, with or without patches | M | Any | overnight |
| KORHON | USA, Boston, Mass. | 15 week RCT of patches and exercise for cessation in smokers of 5+/day | F | 18-55 |  |
| KUME | Japan, Fuchu, Tokyo | 3 year community study based on annual health checks | B | 21-95 | overnight |
| KUSHIM | Japan, Hiroshima | 5 year workers follow-up study | M | 30-59 | overnight^e^ |
| LEE | Korea, Seoul | 2 month cessation study in nicotine dependent smokers, hospital workers | M | 28-52 | overnight |
| LUDVIK | Iceland, Reykjavik | 3 month RCT of nasal spray for cessation in smokers of 1+/day | B | 21-68 | overnight |
| MASARE | Australia, Perth | 6 month cessation study | B | Any | ^[[9]](#endnote-9)^ |
| MOFFA1 | USA, Tallahassee, Fla. | 60 day cessation study in smokers of 20+/day for 5 y | F | Any | overnight^e^ |
| MOFFA2 | USA, Tallahassee, Fla. | 30 day cessation study in smokers of 20+/day for 5y | B | Any | overnight^e^ |
| MOFFA3 | USA, Tallahassee, Fla. | 77 day RCT of patches for cessation, not using other tobacco products, with community controls | B | Any | overnight |
| NIAURA | USA, Providence, RI | 12 week RCT of exercise for cessation in smokers of 3y | F | Any | overnight |
| NILSSO | Sweden, southern, 20 centres | 4 month RCT of group counselling with gum and patches in smokers of >10/day for 10y | B | 30-60 | overnight^e^ |
| NORREG | Denmark, Copenhagen | 1 year RCT of ephedrine and caffeine for cessation in smokers of 10+/day for 3y | B | 25-65 |  |
| PRIEME | Denmark, Copenhagen | 26 week RCT of patches for cessation in smokers of 15+/day for 1y | B | 35-65 | overnight |
| PULS | Germany, Goettingen | 5 week cessation study with optional NRT | B | Any | ^[[10]](#endnote-10)^ |
| QUENSE | Sweden, Lund | 2 week cessation study in cigarette or pipe smokers for 5y | M | 26-42 | overnight |
| RABKIN | Canada, Vancouver | 2-3 month RCT of behaviour modification and hypnosis for cessation | B | Any | overnight |
| RAHILL | USA, Boston Mass. | 30 year community study based on 3-yearly examinations (with HDL-C from 1981) | M | 21-81 | yes (unspecified) |
| RICHAR | France, Lille | 3-12 week cessation study | B | Any | overnight |
| SHENNA | UK, London | 4 year study based on periodic screening for executives | M | ≤61 | overnight |
| STAMFO | USA, Louisville, Ky. | 48 day cessation study with 1 year follow-up in smokers of 20+/day for 5y | F | Any | overnight^e^ |
| STUBBE | Sweden, Lund | 6 week cessation study in smokers of 17-30/day for 12+y | M | 32-50 | overnight |
| SUWAZO | Japan, Chiba | 15 year study based on annual health checks for steel workers^[[11]](#endnote-11)^ | M | Any | 30 minutes |
| SWANK | USA, Louisville, Ky. | 7 week cessation study in smokers of 20+/day for 12y | F | 21-45 | overnight^[[12]](#endnote-12)^ |
| TAMURA | Japan, nationwide | 4 year follow-up study of intervention on lifestyle factors in factory workers | M | 19-69 | yes (unspecified)^[[13]](#endnote-13)^ |
| TERRES | Germany, Hamburg | 24 week study of gum for cessation in smokers of 20+/day for 5y | B | Any | overnight^l^ |
| TONSTA | Norway, Oslo | 1 year RCT of buproprion for cessation in smokers of 10+/day | B | 18+ | overnight |
| VANDEN | The Netherlands, Nijmegen | 1 year cessation study in smokers of 5+/day for 5y | M | Any | overnight |
| YAMAMO | Japan, Nagoya | 3 year observational study based on annual health checks in office workers | M | 30-69 | yes (unspecified) |
| YEH | USA, NC, Miss., Minn., Md | 5 year community follow-up study | B | 45-64 | overnight |
| YOON | Korea, Seoul | 12 year observational study based on subjects attending for at least 2 health checks | M | 30+ | overnight |
| ZHANG | China, Beijing | 3-6 month cessation study with patches | M | 21-69 |  |

## Table A1-2 Study design – aspects of diet exercise and weight

| Study REF | Diet/exercise/weight/risk factor^[[14]](#endnote-14)^ criteria for participation | Diet/exercise aspects during study |
| --- | --- | --- |
| ALLEN |  |  |
| BASLER | smokers with additional CHD risk factors | Advice given on improved diet and exercise, with emphasis on prevention of weight gain |
| BURNET |  |  |
| ELIAS1 | normal weight | Subjects were asked not to change diet or physical activity |
| ELIAS2 |  |  |
| FEHER |  |  |
| FERRAR | obese | Subjects ate a controlled diet for 2 days prior to a fat biopsy, but otherwise no control |
| FORTMA |  | Community health education program |
| GEPNER |  |  |
| GERACE | screened to be at high coronary risk, but those with highest cholesterol, BP, BMI at baseline were excluded | Dietary and exercise advice were part of the study intervention program |
| GREEN |  |  |
| HAUSTE |  |  |
| IINO | diabetics |  |
| KONDO |  |  |
| KORHON | little exercise | 2 x 2 factorial randomized trial of exercise setting and level of exercise counselling |
| KUME |  |  |
| KUSHIM |  |  |
| LEE |  | Subjects were instructed not to change their eating habits, and not to drink more than 5 standard alcoholic drinks/week and one cup of coffee/day |
| LUDVIK |  |  |
| MASARE |  |  |
| MOFFA1 | sedentary with little exercise |  |
| MOFFA2 | sedentary with little exercise | Diet and exercise were briefly discussed, but no special instructions or recommendations were provided to encourage or discourage subjects from modifying their diets or activity levels |
| MOFFA3 |  | Subjects were encouraged to act naturally in terms of quantity of food consumed, but were instructed not to make qualitative changes in diet or other lifestyle changes (e.g. amount of physical activity, alcohol consumption) |
| NIAURA | little exercise | Exercise advice was part of the study intervention program |
| NILSSO |  | No advice was given on diet or weight maintenance |
| NORREG | BMI>20 and wanting to avoid weight gain | Subjects involved in competition sports were excluded. The cessation program included advice on diet and preventing weight gain |
| PRIEME |  |  |
| PULS |  |  |
| QUENSE | not doing regular sport | Diet controlled to be the same in cessation period as in 2 weeks pre-cessation |
| RABKIN |  |  |
| RAHILL |  |  |
| RICHAR |  |  |
| SHENNA | normal weight | The authors “endeavoured to use cases showing no net dietary differences with respect to smoking behaviour” |
| STAMFO | sedentary with stable weight | Subjects were asked not to change diet or physical activity |
| STUBBE | sedentary | Participants were encouraged not to change dietary or physical habits |
| SUWAZO |  |  |
| SWANK |  | Feedback from dietary analysis assisted participants to maintain dietary consistency |
| TAMURA |  | Diet and exercise advice or teaching materials were part of the study intervention program |
| TERRES |  |  |
| TONSTA |  |  |
| VANDEN | BMI ≤30 |  |
| YAMAMO |  |  |
| YEH |  |  |
| YOON |  |  |
| ZHANG |  |  |

Blank cell indicates no relevant criteria

## Table A1-3 Medical criteria for population eligibility

| ALLEN | Generally healthy as determined by medical history, physical exam, routine laboratory tests (screening lab values could not exceed normal range by 20%) and a 12-lead electrocardiogram. If female, they needed an acceptable method of birth control. Patients were not enrolled if they had a history of alcohol abuse in the past year, or used psychotropic drugs, steroids, or antihistamines. Patients were also excluded if they had a history of myocardial infarction, angina pectoris, sustained or episodic cardiac arrhythmias that could be aggravated by nicotine, Buerger's disease, Prinzmetal variant angina, symptomatic peripheral vascular disease, insulin-dependent diabetes, or other medical conditions which the investigator deemed inappropriate for patient participation |
| --- | --- |
| BASLER | Has had a diagnosis of a CHD risk factor |
| BURNET | To have menstruated within the past 3 months, to have no surgical menopause, to have a diastolic BP less than 100 mm Hg, and not to be taking lipid-lowering drugs, insulin, thyroid medication, estrogens, antihypertensive drugs, or psychotropic drugs |
| ELIAS1 | Normal weight (BMI<27), normotensive BP<150/95), taking no chronic medication |
| ELIAS2 | No diabetes mellitus, blood pressure <=160/95 mmHg. Not pregnant or breast feeding, receiving no psychiatric care or medication. No history of alcohol abuse. No concomitant chronic medication with known metabolic effects |
| FERRAR | No evidence of diabetes, hypertension, hyperlipidemia, cancer, liver, renal or hematological diseaase. Not taking any medication |
| GEPNER | Not currently taking bupropion or having a psychosis or schizophrenia diagnosis. No medical contraindications for any of the study medications, including high alcohol consumption, a history of seizure, high blood pressure (>160/100 mmHg), bipolar disorder, an eating disorder, a recent cardiac event or allergies to any of the medications. Not currently pregnant or lactating. |
| GERACE | In the top 10-15% of a risk score distribution based on the Framingham Heart Study. Risk score based on cigarette smoking, serum cholesterol and blood pressure but excluding if serum cholesterol >350 mg/dL or diastolic BP >115. No history of heart attack, diabetes or angina. No treatment with guanethidine, hydralazine, insulin, oral hypoglycaemic agents or lipid-lowering agents. Body weight <150% of desirable |
| GREEN | Did not start medication during the study period |
| HAUSTE | No MI in last 3 months, unstable angina pectoris, hypertension requiring medical treatment, liver or kidney disease, chronic infectious disease or metabolic disease, alcohol or drug problems. Not using medication that would interfere with the trial parameters |
| IINO | No change to medication over the study period |
| KONDO | No medications including statins, antidiabetic drugs or antihypertensive drugs |
| KORHON | Nonpregnant, free of CVD, no active and severe psychiatric illness, insulin-dependent diabetes mellitus or skin condition contraindicating patch use |
| LEE | No taking of illegal drugs or other medication. No medical or physical disorders other than nicotine dependence. No family history of psychiatric illness or medical problems |
| LUDVIK | No recent MI, severe allergy, current alcohol or drug abuse or pregnancy or breastfeeding |
| MASARE | Not taking anti-hypertensive medication |
| MOFFA1 | Not using oral contraceptives or lipid altering medications. Free of known heart disease and asymptomatic |
| MOFFA2 | Not using oral contraceptives or lipid altering medications, including beta-adrenergic blocking agents. Free from known heart disease and asymptomatic. Not post-menopausal |
| MOFFA3 | Healthy as determined by medical questionnaire |
| NIAURA | No CHD, substance abuse or orthopedic problems. Not using medications that could affect serum lipids, including oral contraceptives or exogenous estrogens |
| NILSSO | Free of chronic disease |
| NORREG | Free from cardiovascular disease, previous myocardial infarction or heart failure, hyperthyroidism, gastric or duodenal ulcers within the last 3 months, pregnancy or breast-feeding, daily use of psychotropic drugs (including anxiolytics), daily alcohol consumption of more than 3 drinks per day, acute medical disease, hypertension, low body weight (body mass index <20 kg/m2), and intake of ephedrine-caffeine combination drugs during the last 2 years |
| PRIEME | No known disease, not pregnant or breast-feeding. Not taking drugs including contraceptives and antioxidants |
| QUENSE | No medication during the observation period |
| RAHILL | No SBP >140mm Hg, DBP > 90mm Hg, diabetes mellitus, CHD or cancer at enrollment |
| RICHAR | Triglycerides <= 4.6 mmol/L |
| SHENNA | Body weight <=120% of ideal. Fasting plasma glucose <120mg% or fasting mean total plasma cholesterol <270mg%. Not using prescribed drugs other than aspirin, dipyridamole, glyceryl trinitrate, disopyramide or benzodiazepines |
| STAMFO | Taking no medication with a stable body weight |
| STUBBE | Taking no medication and feeling well |
| SUWAZO | Not treated for diabetes, cardio- and cerebro- vascular disease, hyperlipidemia and/or malignant neoplasm |
| SWANK | Subjects were assessed by medical history questionnaire, cardiovascular risk factor profile, electrocardiogram, blood pressure, skinfold assessment, blood lipid profile and glucose screening for diabetes but criteria for inclusion in the study are not stated |
| TAMURA | Not taking antihypertensive or lipid-lowering agents |
| TERRES | Apparently healthy. No aspirin or nonsteroidal antirheumatic drugs |
| TONSTA | No seizure, current diagnosis of major depressive episode or history of panic disorder. No psychosis, bipolar disorder or eating disorders. No pregnancy or lactation, alcohol abuse or drugs other than nicotine. No use of psychoactive drugs with the week before enrolment or bupropion within the month before enrolment. No current use of other smoking-cessation treatments |
| VANDEN | No cardiovascular disease, no irregular heart rhythm disturbances, no use of antihypertensive, lipid or glucose lowering medication or hormonal medication, BMI<=30kgm-2, no hypertension (SBP >160 mmHg and/or DBP >95 mmHg, no diabetes (history or non-fasting glucose >11.1), no hypercholesterolaemia (non-fasting total plasma cholesterol >6.5 mmolL) and an ankle-arm index >0.80 |
| YEH | No pre-existing diabetes, asthma, chronic lung disease or prevalent heart disease, no incident diabetes during study period |
| ZHANG | No cardiovascular disease, arrhythmia, abnormal thyroid function, liver & kidney dysfunction or other disease |

No medical criteria were specified for the remaining studies: FEHER, FORTMA, KUME, KUSHIM, PULS, RABKIN, YAMAMO, YOON

## Table A1-4 Methods for determination of HDL-C values

| BASLER | Plasma measurements were taken using commercially available kits (Reflotron, Boehringer Mannheim) |
| --- | --- |
| ELIAS1 | All blood samples were drawn in appropriate tubes, kept on ice until centrifuged and stored at -20°C. Serum HDL-cholesterol was measured by the phosphotungstic acid-magnesium chloride precipitation method |
| ELIAS2 | Cholesterol was measured by enzymatic methods with the concentration of HDL-C being measured by the phosphotungstic/magnesium chloride precipitation method |
| FEHER | HDL was isolated by selective precipitation of apo-B lipoproteins with dextran sulphate and magnesium chloride. Total cholesterol (-C) and HDL-C were analysed enzymatically using Boehringer reagents on a Centrificem centrifugal analyser. Venous blood samples were taken in the evening with minimal haemostasis and with the subject sitting |
| FERRAR | Venous blood samples were transferred into chilled tubes containing 1g EDTA/l blood, and plasma was separated by centrifugation by 4°C for 15 min at 2000 g |
| FORTMA | Venous blood was obtained while seated. Refrigerated plasma samples were analysed fresh within a week by the methods of the Lipid Research Clinic Program |
| GEPNER | Fasting blood samples were obtained by venipuncture and refrigerated. Plasma aliquots were isolated by centrifugation and frozen at -70°C. Samples underwent nuclear magnetic resonance spectroscopic lipoprotein analysis |
| GERACE | Serum cholesterol was determined by automated methods. The cholesterol content of each lipoprotein fraction was estimated after heparin/manganese precipitation |
| GREEN | Venous blood samples were drawn in vacuum tubes without additive and with EDTA, with the subject sitting. Cholesterol was determined by the enzymatic colour method (Lancer) |
| HAUSTE | Blood samples were collected from the cubital vein. Samples were centrifuged and plasma stored in a freezer. HDL-C was measured photometrically |
| IINO | HDL-C was measured by enzymatic methods |
| KUSHIM | HDL-C analysis was carried out within 24 hours of blood collection using the heparin magnesium method |
| LEE | Enzymatic techniques using a Hitachi 7600-110 analyzer |
| LUDVIK | Venous blood samples were drawn after overnight fasting. Samples for estimating HDL cholesterol were centrifuged within 1 hour of collection and the separated serum kept frozen. HDL cholesterol was measured by an enzymatic colorimetric test with the cholesterol esterase, cholesterol oxidase and POD catalysed indicatorreactin method, after precipitating the other lipoproteins with phosphotungstic acid, 1.4 mmol/L, plus magnesium chloride, 8.6 mmol/L |
| MASARE | Samples were collected after 20 minutes of supine rest. HDL was assayed on a heparin-manganese chloride supernatant (final manganese concentration 0.046 mol/L) with a coefficient of variation of 3.2 |
| MOFFA1 | Blood samples were drawn from an antecubital vein following a 12-h abstinence from smoking. HDL-C was measured by precipitating all other cholesterol fractions with phosphotungstate-magnesium leaving a supernatant which was measured for cholesterol content |
| MOFFA2 | Blood was drawn from an antecubital vein. HDL-C was isolated by sequential polyanionic double precipitation using heparin-manganese chloride |
| MOFFA3 | Blood was sampled from an antecubital vein. HDL was separated from lipoproteins containing apolipoprotein B by a precipitation technique using heparin-manganese |
| NIAURA | HDL-C was estimated after precipitation of lower density lipoproteins |
| NILSSO | Blood samples were drawn and serum separated. Samples were then stored at -22°C until analysis. HDL-C was determined by routine analysis |
| NORREG | Venous blood samples were taken in the afternoon. HDL-C was determined by standard laboratory methods |
| PULS | HDL-C was determined enzymatically |
| QUENSE | Blood samples were drawn between 8 and 9 a.m. The lipid components of HDL were determined in the supernatant obtained after precipitation of VLDL and LDL by MgCl2 and dextran sulphate |
| RABKIN | HDL-C was analysed by the Beckman Lipoprotein profiling system after manganese heparin precipitation |
| RICHAR | Venous blood was drawn into EDTA tubes by venipuncture. Cholesterol was determined in the HDL-containing supernatant after phosphotungstate/magnesium chloride precipitation (4.4% and 2.7%) |
| SHENNA | Blood was collected from semi-recumbant patients after a 20 minute rest via an ante-cubital vein cannula. HDL-C was measured by the method of Burnstein et al. Lipoprotein-poly-anion metal interactions, Adv. Lipid. Res., 11 (1973) 67 |
| STAMFO | Venous blood from a superficial vein. HDL-C was measured by precipitating all other cholesterol fractions with heparin and manganese |
| STUBBE | Enzymatic methods |
| SWANK | After a 48-hour abstinance from alcohol and 20 minutes of quiet sitting a resting blood sample was drawn and allowed to clot and serum collected. Serum samples were stored at -4°C and analysed weekly. HDL-C was measured by enzymatic methods |
| TERRES | Blood was collected from a large antecubital vein without venous occlusion and HDL-cholesterol was measured by standard methods |
| TONSTA | HDL-C was measured using enzymatic methods adapted to Cobas Integra |
| VANDEN | HDL was determined with the phosphotungstate/Mg2+ method |
| YAMAMO | Venous blood was analysed by an in-house chemical autoanalyzer. Participants were asked not to take any drugs for at least 14 hours before blood sampling |
| YEH | Minimally traumatic venipuncture. Measured after dextran-magnesium precipitation |
| YOON | Measured by standard techniques in a central, certified laboratory from venous blood samples collected the same morning from subjects who had fasted for at least 12 hours |

The method was unspecified for the remaining studies: ALLEN, BURNET, KONDO, KORHON, KUME, PRIEME, RAHILL, SUWAZO, TAMURA, ZHANG.

## Table A1-5 Baseline physiological parameters

| Study REF | Sex or strata^[[15]](#endnote-15)^ | Smoking group^[[16]](#endnote-16)^ | HDL-C  (mmol/l) | LDL-C  (mmol/l) | TG  (mmol/l) | Weight  (kg) | BMI | SBP  (mmHg) | DBP  (mmHg) |
| --- | --- | --- | --- | --- | --- | --- | --- | --- | --- |
|  |  |  |  |  |  |  |  |  |  |
| ALLEN | B | Q | 1.164 | 3.703 | 1.646 | 72.5 |  | 121.4 | 75.9 |
| BASLER | B | Q | 1.257 |  |  | 75.8 |  | 136.7 | 79.9 |
|  |  | C | 1.304 |  |  | 73.4 |  | 131.4 | 79.3 |
| BURNET | F | Q |  |  |  |  |  |  |  |
|  |  | N |  |  |  |  |  |  |  |
|  |  | C |  |  |  |  |  |  |  |
| ELIAS1 | M | Q | 1.000 | 3.900 | 1.300 | 78.2 | 23.5 | 119.0 | 69.0 |
| ELIAS2 | B | Q | 1.160 | 3.780 |  |  |  |  |  |
| FEHER | B | Q | 1.470 |  |  |  |  |  |  |
| FERRAR | F | Q | 1.010 | 2.350 | 1.360 | 95.1 |  |  |  |
| FORTMA | B | Q | 1.355 | 3.220 | 1.467 |  | 24.3 | 124.0 | 76.9 |
| GEPNER | B | Q | 1.099 | 3.108 | 1.578 | 84.6 | 29.0 | 120.8 | 75.1 |
| GERACE | M | Q |  |  |  |  |  |  |  |
|  |  | C |  |  |  |  |  |  |  |
| GREEN | M | Q | 1.050 | 3.670 | 1.962 |  |  | 125.5 | 79.4 |
|  |  | C | 1.068 | 3.341 | 1.714 |  |  | 123.0 | 76.8 |
| HAUSTE | M | Q | 1.009 |  |  |  |  |  |  |
|  |  | C | 1.145 |  |  |  |  |  |  |
| KONDO | M | Q | 1.560 |  | 1.550 |  | 22.2 | 117.6 |  |
| KORHON | F | Q | 1.485 | 3.026 | 1.423 | 75.0 | 28.4 | 114.0 | 73.4 |
| KUSHIM | M | Q |  |  |  |  |  |  |  |
|  |  | N |  |  |  |  |  |  |  |
|  |  | C |  |  |  |  |  |  |  |
| LEE | M | Q | 1.578 | 3.153 | 1.611 | 68.6 | 23.9 |  |  |
| LUDVIK | B | Q | 1.290 | 4.000 | 1.380 |  |  |  |  |
| MASARE | M | Q | 1.100 |  | 1.120 | 74.5 |  |  |  |
|  | F | Q | 1.430 |  | 0.850 | 58.8 |  |  |  |
| MOFFA1 | F-P | Q | 1.329 |  | 0.898 | 58.0 |  |  |  |
|  | F-R | Q | 1.291 |  | 0.854 | 58.9 |  |  |  |
|  | F | N | 1.547 |  | 0.915 | 62.4 |  |  |  |
|  | F | C | 1.229 |  | 0.930 | 57.1 |  |  |  |
| MOFFA2 | M | Q | 0.940 | 3.460 | 1.660 | 77.9 |  |  |  |
|  |  | N | 1.146 | 3.370 | 1.380 | 79.5 |  |  |  |
|  |  | C | 0.905 | 3.650 | 1.430 | 76.7 |  |  |  |
|  | F | Q | 1.160 | 3.160 | 1.160 | 58.6 |  |  |  |
|  |  | N | 1.437 | 3.170 | 1.010 | 61.8 |  |  |  |
|  |  | C | 1.224 | 3.030 | 1.200 | 62.8 |  |  |  |
| MOFFA3 | M | Q | 0.931 |  |  | 73.5 |  |  |  |
|  |  | N | 1.172 |  |  | 75.9 |  |  |  |
|  | F | Q | 1.115 |  |  | 65.3 |  |  |  |
|  |  | N | 1.397 |  |  | 65.7 |  |  |  |
| NIAURA | F-E | Q | 1.164 | 3.362 | 1.186 |  | 28.1 |  |  |
|  | F-C | Q | 1.110 | 2.974 | 0.869 |  | 25.8 |  |  |
| NILSSO | B | Q | 1.000 | 3.800 | 1.600 | 74.5 |  | 120.5 | 74.2 |
|  |  | C | 1.000 | 3.700 | 1.300 | 74.6 |  | 117.2 | 72.8 |
| NORREG | B | Q | 1.300 |  |  |  |  |  |  |
| PRIEME | B-P | Q | 1.340 | 3.560 | 1.180^[[17]](#endnote-17)^ | 75.0 | 25.2 |  |  |
|  | B-R | Q | 1.378 | 4.312 | 1.274^[[18]](#endnote-18)^ | 75.0 | 24.3 |  |  |
|  | B | C | 1.390 | 3.960 | 1.160^c^ | 75.2 | 24.3 | 120.0^c^ | 80.0^c^ |
| PULS | B | Q | 1.474 | 3.439^c^ |  | 72.0 |  |  |  |
| QUENSE | M | Q | 1.010 | 2.970 | 1.090 |  |  |  |  |
| RABKIN^[[19]](#endnote-19)^ | M | Q | 1.009 | 2.922 | 1.400 | 69.2 | 24.0 | 118.0 | 81.0 |
|  |  | C | 1.060 | 2.767 | 1.716 | 66.0 | 20.0 | 119.0 | 81.0 |
|  | F | Q | 1.267 |  |  |  |  |  |  |
|  |  | C | 1.267 |  |  |  |  |  |  |
| RAHILL | M | Q |  |  |  |  |  |  |  |
|  |  | N |  |  |  |  |  |  |  |
| RICHAR | B | Q | 1.370 | 3.380 | 1.370 |  | 23.7 |  |  |
| SHENNA | M | Q | 1.241 |  | 1.485 | 79.2 |  |  |  |
| STAMFO | F-P | Q | 1.345 |  | 1.106 |  |  |  |  |
| STAMFO | F-R | Q | 1.267 |  | 0.903 |  |  |  |  |
| STUBBE | M | Q | 0.820 |  | 0.200 |  |  |  |  |
| SUWAZO | M | Q | 1.348 |  |  | 67.3 | 23.5 | 125.5 | 77.1 |
|  |  | C | 1.332 |  |  | 67.1 | 23.5 | 128.1 | 78.2 |
| SWANK | F | Q | 1.337 |  |  | 62.7 |  |  |  |
|  |  | C | 1.353 |  |  | 70.9 |  |  |  |
| TAMURA | M-i | Q | 1.430 |  |  | 67.2 | 22.7 | 117.5 | 72.0 |
|  | M-ii | Q | 1.353 |  |  | 66.6 | 23.1 | 116.7 | 72.3 |
|  | M-iii | Q | 1.448 |  |  | 65.6 | 22.8 | 113.4 | 69.9 |
|  | M | C | 1.397 |  |  | 66.1 | 22.8 | 117.0 | 71.4 |
| TERRES | B | Q | 1.544 | 3.473 | 1.106^c^ | 68.7 | 22.3 | 119.5 | 79.8 |
| TONSTA | B | Q | 1.460 | 3.770 | 1.580 |  |  |  |  |
| VANDEN | M | Q | 1.200 | 3.920 | 1.330 | 78.0 |  |  |  |
|  |  | N | 1.280 | 3.700 | 1.130 | 77.9 | 24.1 | 136.0 | 83.0 |
|  |  | C | 1.240 | 3.650 | 1.270 | 77.0 | 23.8 | 130.0 | 79.0 |
| YEH | B | Q | 1.300 |  | 1.500 | 73.9 |  | 117.0 | 71.0 |
|  |  | N | 1.400 |  | 1.320 | 77.3 |  | 120.0 | 74.0 |
|  |  | C | 1.320 |  | 1.410 | 72.6 |  | 117.0 | 70.0 |
| YOON | M-L | Q |  |  |  |  |  |  |  |
|  | M-H | Q |  |  |  |  |  |  |  |
|  | M | C | 1.239 | 3.173 | 1.874 | 69.6 | 24.2 | 124.4 | 77.8 |
| ZHANG | M | Q | 1.154 | 3.171 | 1.524 |  |  | 120.1 | 78.4 |

Rows in this table correspond to the main data set analysed, with continuing smoker and non smoker data shown on a grey background. Blank cell indicates data not available. Data are means except where indicated otherwise.

## Table A1-6 Baseline smoking

| Study REF | Sex or strata^[[20]](#endnote-20)^ | Smoking group^[[21]](#endnote-21)^ | Product/ definition^[[22]](#endnote-22)^ | N cigs^[[23]](#endnote-23)^ | Quitting validated^[[24]](#endnote-24)^ |
| --- | --- | --- | --- | --- | --- |
|  |  |  |  |  |  |
| ALLEN | B | Q | Cigs only |  | Yes (CO) |
| BASLER | B | Q | Cigs +/- | ^[[25]](#endnote-25)^ | Yes (CO) |
|  |  | C | Cigs +/- | ^[[26]](#endnote-26)^ |  |
| BURNET | F | Q | Cigs +/- |  | No mention |
|  |  | N | Non Cigs |  |  |
|  |  | C | Cigs +/- |  |  |
| ELIAS1 | M | Q | Cigs +/- |  | Yes (COT) at wk8, No at wk 35 |
| ELIAS2 | B | Q | Cigs +/- | 21.5 | Yes (CO) |
| FEHER | B | Q | Cigs +/- | 20.0^[[27]](#endnote-27)^ | Yes (COT, N) |
| FERRAR | F | Q | Any |  | Yes (CO) |
| FORTMA | B | Q | Cigs +/- | 14.0 | Yes (CO, T) |
| GEPNER | B | Q | Cigs only | 20.0 | Yes (CO) |
| GERACE | M | Q | Cigs +/- |  | Yes (T) |
|  |  | C | Cigs +/- |  |  |
| GREEN | M | Q | Cigs +/- |  | No mention |
|  |  | C | Cigs +/- |  |  |
| HAUSTE | M | Q | Cigs only |  | Yes (CO) |
|  |  | C | Cigs only |  |  |
| KONDO | M | Q | Cigs +/- | ^[[28]](#endnote-28)^ | No |
| KORHON | F | Q | Cigs +/- | 15.3 | Yes (CO) |
| KUSHIM | M | Q | Cigs +/- |  | No mention |
|  |  | N | Nev Cigs |  |  |
|  |  | C | Cigs +/- |  |  |
| LEE | M | Q | Any | 19.3 | Yes (COT) |
| LUDVIK | B | Q | Cigs +/- |  | Yes (CO) |
| MASARE | M | Q | Any | 23.3^[[29]](#endnote-29)^ | Yes (T) |
|  | F | Q | Any | ^j^ | Yes (T) |
| MOFFA1 | F-P | Q | Cigs +/- | 31.0 | No mention |
|  | F-R | Q | Cigs +/- | 29.0 | No mention |
|  | F | N | Nev Any |  |  |
|  | F | C | Cigs +/- | 29.0 |  |
| MOFFA2 | M | Q | Cigs +/- | 44.3 | No |
|  |  | N | Nev Cigs |  |  |
|  |  | C | Cigs +/- | 36.3 |  |
|  | F | Q | Cigs +/- | 27.0 | No |
|  |  | N | Nev Cigs |  |  |
|  |  | C | Cigs +/- | 29.0 |  |
| MOFFA3 | M | Q | Cigs only | 29.2 | No mention |
|  |  | N | Nev Any |  |  |
|  | F | Q | Cigs only | 28.6 | No mention |
|  |  | N | Nev Any |  |  |
| NIAURA | F-E | Q | Any | 19.0^[[30]](#endnote-30)^ | Yes (CO) |
|  | F-C | Q | Any |  | Yes (CO) |
| NILSSO | B | Q | Cigs +/- | 21.7 | Yes (COT, N) |
|  |  | C | Cigs +/- | 18.8 |  |
| NORREG | B | Q | Cigs +/- |  | Yes (CO) |
| PRIEME | B-P | Q | Cigs +/- | 20.0^h^ | Yes (CO) |
|  | B-R | Q | Cigs +/- | 20.0^h^ | Yes (CO) |
|  | B | C | Cigs +/- | 23.0^[[31]](#endnote-31)^ |  |
| PULS | B | Q | Cigs +/- | 20.3 | Yes (CO) |
| QUENSE | M | Q | Any | 16.0^[[32]](#endnote-32)^ | Yes (COHb) |
| RABKIN | M | Q | Cigs +/- | 26.1^j^ | Yes (T) |
|  |  | C | Cigs +/- | 32.0^j^ |  |
|  | F | Q | Cigs +/- | ^j^ | Yes (T) |
|  |  | C | Cigs +/- | ^j^ |  |
| RAHILL | M | Q | Any |  | No mention |
|  |  | N | Non Any |  |  |
| RICHAR | B | Q | Cigs +/- | 22.2 | Yes (CO) |
| SHENNA | M | Q | Any |  | No mention |
| STAMFO | F-P | Q | Cigs +/- |  | No mention |
| STAMFO | F-R | Q | Cigs +/- |  | No mention |
| STUBBE | M | Q | Cigs +/- | 21.0 | Yes (COHb) |
| SUWAZO | M | Q | Any |  | No |
|  |  | C | Any |  |  |
| SWANK | F | Q | Cigs +/- |  | Yes (COHb) |
|  |  | C | Cigs +/- |  |  |
| TAMURA | M-i | Q | Any | 16.7 | No |
|  | M-ii | Q | Any | 20.2 | No |
|  | M-iii | Q | Any | 17.3 | No |
|  | M | C | Any | 21.0 |  |
| TERRES | B | Q | Cigs +/- | 26.7 | No |
| TONSTA | B | Q | Cigs only | 20.0 | Yes (CO) |
| VANDEN | M | Q | Cigs +/- |  | Yes (C) |
|  |  | N | Non Any^[[33]](#endnote-33)^ |  |  |
|  |  | C | Cigs +/- |  |  |
| YEH | B | Q | Cigs +/- |  | No |
|  |  | N | Nev Any |  |  |
|  |  | C | Cigs +/- |  |  |
| YOON | M-L | Q | Cigs +/- |  | No |
|  | M-H | Q | Cigs +/- |  | No |
|  | M | C | Cigs +/- |  |  |
| ZHANG | M | Q | Any |  | Yes (CO) |

Rows in this table correspond to the main data set analysed, with continuing smoker and non smoker data shown on a grey background

1. RCT = randomised controlled trial. *x*+/day refers to number of cigarettes smoked. y is duration of smoking in years. “other tobacco products” refers to pipe, cigar and smokeless tobacco [↑](#endnote-ref-1)
2. B = both sexes, F = females, M = males [↑](#endnote-ref-2)
3. HDL-C measurement taken after fasting. Any requirement to abstain from smoking before measurement is mentioned in further footnotes [↑](#endnote-ref-3)
4. Only at one of 9 centres ALLEN [↑](#endnote-ref-4)
5. Also after abstinence from smoking ELIAS1, FERRAR, KUSHIM, MOFFA1, MOFFA2, NILSSO, STAMFO [↑](#endnote-ref-5)
6. ^f^ Subjects were studied 2 weeks before and 2 weeks after stopping smoking FEHER [↑](#endnote-ref-6)
7. Results refer to 2 cities with intervention programme and 2 control cities combined FORTMA [↑](#endnote-ref-7)
8. Intervention comprised BP medication, and dietary, exercise and smoking cessation advice GERACE [↑](#endnote-ref-8)
9. Measurements taken after abstinence from smoking for at least 1 hour MASARE [↑](#endnote-ref-9)
10. Measurements taken after abstinence from smoking for at least 90 minutes PULS [↑](#endnote-ref-10)
11. Quitters were those who reported smoking at one examination followed by three successive nonsmoking reports. One set of four successive smoking reports was randomly selected for smoking controls. SUWAZO [↑](#endnote-ref-11)
12. Also after 2 hours smoking and gum abstinence SWANK, TERRES [↑](#endnote-ref-12)
13. For approximately 80% of participants. TAMURA [↑](#endnote-ref-13)
14. Studies restricted to subjects with traditional coronary risk factors are mentioned here. Other studies which exclude such subjects are listed in Table A1-3 [↑](#endnote-ref-14)
15. B = both sexes, F = females, M = males; L=low weight gain (<1.3 kg), H=high weight gain (≥1.3 kg); P = persistent quitter, R = resumed smoking before end of study; E = exercise training intervention, C = control group, i = quit <1yr, ii = quit 1-2 years, iii = quit ≤2yrs [↑](#endnote-ref-15)
16. Q = Quitter, C = continuing smoker, N = never or non smoker. C and N rows are shown with grey background [↑](#endnote-ref-16)
17. Median [↑](#endnote-ref-17)
18. Approximate estimate (medians combined over groups) PRIEME [↑](#endnote-ref-18)
19. Except for HDL-C which was reported separately, results shown against males are for the sexes combined RABKIN [↑](#endnote-ref-19)
20. B = both sexes, F = females, M = males; L=low weight gain (<1.3 kg), H=high weight gain (≥1.3 kg); P = persistent quitter, R = resumed smoking before end of study; E = exercise training intervention, C = control group, i = quit <1yr, ii = quit 1-2 years, iii = quit ≤2yrs [↑](#endnote-ref-20)
21. Q = Quitter, C = continuing smoker, N = never or non smoker. C and N rows are shown with grey background [↑](#endnote-ref-21)
22. Any = any product (cigarettes, cigars or pipes), Cigs = cigarettes, Cigs +/− = cigarettes with or without other products, Cigs only = only cigarettes. Nev = never, Non = not current [↑](#endnote-ref-22)
23. Blank cell on Q and C rows indicate data not available. Data are means except where indicated otherwise. [↑](#endnote-ref-23)
24. No = quitting not validated, Yes = quitting validated, with chemical(s) used indicated by COT = cotinine, CO = carbon monoxide, COHb = carboxyhaemoglobin, N = nicotine, T = thiocyanate [↑](#endnote-ref-24)
25. 65% smoked >20 cigs/day BASLER (quitters) [↑](#endnote-ref-25)
26. 60% smoked >20 cigs/day BASLER (continuing smokers) [↑](#endnote-ref-26)
27. Median FEHER, PRIEME [↑](#endnote-ref-27)
28. 33% smoked 20+ cigs/day KONDO [↑](#endnote-ref-28)
29. Results shown against males are for the sexes combined MASARE, RABKIN [↑](#endnote-ref-29)
30. Result shown against the first stratum is for the whole study, and stated not to differ by intervention group NIAURA [↑](#endnote-ref-30)
31. Approximate estimate (median combined over groups) PRIEME [↑](#endnote-ref-31)
32. 10 cigarettes smokers, mean 16 cigs/day, and 2 pipe smokers, mean 1.1 g/day QUENSE [↑](#endnote-ref-32)
33. Has not smoked for 5 years VANDEN [↑](#endnote-ref-33)
