# Supplementary material for: The effect of quitting smoking on HDL-cholesterol - a review based on within-subject changes
Source: Biomark Res. 2013 Sep 13;1:26. doi: 10.1186/2050-7771-1-26 (PMC4177613; doi:10.1186/2050-7771-1-26)
Supplement: Additional file 2 — Changes in HDL-C, bodyweight and BMI. This file presents the data on change in HDL-C, body weight and BMI for each available intra-measurement period, separately for quitters, continuing smokers and never smokers. [file 2050-7771-1-26-S2.docx]

**The effect of quitting smoking on HDL-Cholesterol - A review based on within-subject changes**

# Barbara A Forey, John S Fry, Peter N Lee, Alison J Thornton and Katharine J Coombs

# Additional File 2 – changes in HDL-C, body weight and BMI

Table A2-1 gives the data on change in HDL-C, body weight and BMI in quitters, for each available intra-measurement period, with the main data set shown in the body of the table and all additional stratified or alternative data shown in the footnotes. Equivalent data for continuing smokers are given in Table A2-2, and for never smokers in Table A2-3.

## Table A2-1 Changes in HDL-C (mmol/l), bodyweight (kg) and BMI (kg/m^2^) following quitting

| Study REF | Adjust- ment factors | Sex or strata^[[1]](#endnote-1)^ | Period^[[2]](#endnote-2)^ | Time quit^[[3]](#endnote-3)^ | N^[[4]](#endnote-4)^ | HDL-C | | | Weight^[[5]](#endnote-5)^ | BMI^e^ |
| --- | --- | --- | --- | --- | --- | --- | --- | --- | --- | --- |
|  |  |  |  |  |  | Change | SE | SE Der^[[6]](#endnote-6)^ | Change | Change |
|  |  |  |  |  |  |  |  |  |  |  |
| ALLEN | None | B | 6 |  | 432 | 0.062^[[7]](#endnote-7)^ | 0.019 | P Sig | 2.0 |  |
| BASLER | None | B | 12 | 9 | 55 | 0.041 | 0.035 | SD(Est), N | 1.7 |  |
| BURNET | None | F | av 243 ^[[8]](#endnote-8)^ | 121.5 | 26 | 0.104 | 0.044 | SD, N | 5.2 |  |
|  |  |  | 300 ^[[9]](#endnote-9)^ | 150 | 25 | 0.113 | 0.047 | SD, N | 6.5 |  |
| ELIAS1 | None | M | 8 |  | 17 | 0.200 | 0.063 | SD(Est), N | 2.8 | 0.9 |
|  |  |  | 35 |  | 10 | 0.100 | 0.082 | SD(Est), N |  | 1.5 |
| ELIAS2 | None | B | 16 (8) | 8 | 33 | 0.160 | 0.045 | SD(Est), N |  |  |
| FEHER | None | B | 4 (2) | 2 | 30 | 0.090^[[10]](#endnote-10)^ | 0.048 | SD(Est), N | ^[[11]](#endnote-11)^ |  |
| FERRAR | None | F | 4 |  | 5 | 0.130 | 0.056 | P Sig | 2.6 |  |
| FORTMA | None | B | 156 | 78 | 40 | 0.176^[[12]](#endnote-12)^ | 0.051 | SD, N |  | 0.8 |
| GEPNER | None | B | 52 |  | 334 | 0.062 | 0.012 | SD, N | 4.6 | 1.6 |
| GERACE | None | M | 313 | 156 | 1233 | 0.013 | 0.007 | SD, N | 2.1 |  |
| GREEN | Age | M | 52-209 av 130 | 65 | 68 | 0.044^[[13]](#endnote-13)^ | 0.030 | RNS CI |  | 0.7 |
| HAUSTE | None | M | 4 |  | 51 | 0.133 | 0.036 | SD(Est), N |  |  |
|  |  |  | 8 |  |  | 0.173 | 0.036 | SD(Est), N |  |  |
|  |  |  | 12 |  |  | 0.143 | 0.036 | SD(Est), N |  |  |
|  |  |  | 26 |  |  | 0.147 | 0.036 | SD(Est), N |  |  |
| IINO | None | B | 52 |  | 15 | ^[[14]](#endnote-14)^ |  |  | 1.2 |  |
| KONDO | None | M | 4 |  | 15 | -0.040 | 0.067 | SD(Est), N |  |  |
| KORHON | None | F | 15 | 12 | 58 | 0.044 | 0.034 | SD(Est), N | 1.3 | 0.5 |
| KUME | None | B | 52-156 | 52 | 38 | ^n^ |  |  |  |  |
| KUSHIM | None | M | 261 | 130.5 | 26 | -0.138 | 0.047 | CI |  | 0.8 |
| LEE | None | M | 9 |  | 12 | -0.001 | 0.107 | SD, N | 0.8 | 0.3 |
| LUDVIK | None | B | 13 |  | 46 | 0.150 | 0.074 | P Sig | 3.6 |  |
| MASARE | None | M | 6 |  | 11 | 0.120 | 0.079 | SD(Est), N | 1.7 |  |
|  |  | F |  |  | 15 | 0.010 | 0.067 | SD(Est), N | 1.8 |  |
| MOFFA1 | None | F-P | 4 |  | 12 | 0.147 | 0.075 | SD(Est), N | ^[[15]](#endnote-15)^ |  |
|  |  |  | 9 |  |  | 0.323 | 0.075 | SD(Est), N | 3.6 |  |
|  |  | F-R | 4 |  | 6 | 0.272 | 0.106 | SD(Est), N | 1.2 |  |
| MOFFA2 | None | M | 2 days |  | 7 | 0.099 | 0.099 | SD(Est), N | ^o^ |  |
|  |  |  | 4 days |  |  | -0.007 | 0.099 | SD(Est), N | ^o^ |  |
|  |  |  | 6 days |  |  | 0.021 | 0.099 | SD(Est), N | ^o^ |  |
|  |  |  | 8 days |  |  | 0.028 | 0.099 | SD(Est), N | ^o^ |  |
|  |  |  | 10 days |  |  | 0.000 | 0.099 | SD(Est), N | ^o^ |  |
|  |  |  | 12 days |  |  | 0.028 | 0.099 | SD(Est), N | ^o^ |  |
|  |  |  | 14 days |  |  | 0.043 | 0.099 | SD(Est), N | ^o^ |  |
|  |  |  | 18 days |  |  | 0.043 | 0.099 | SD(Est), N | ^o^ |  |
|  |  |  | 22 days |  |  | 0.035 | 0.099 | SD(Est), N | ^o^ |  |
|  |  |  | 26 days |  |  | 0.043 | 0.099 | SD(Est), N | ^o^ |  |
|  |  |  | 30 days |  |  | 0.035 | 0.099 | SD(Est), N | 1.2 |  |
|  |  | F | 2 days |  | 10 | 0.014 | 0.082 | SD(Est), N | ^o^ |  |
|  |  |  | 4 days |  |  | 0.057 | 0.082 | SD(Est), N | ^o^ |  |
|  |  |  | 6 days |  |  | 0.064 | 0.082 | SD(Est), N | ^o^ |  |
|  |  |  | 8 days |  |  | 0.092 | 0.082 | SD(Est), N | ^o^ |  |
|  |  |  | 10 days |  |  | 0.121 | 0.082 | SD(Est), N | ^o^ |  |
|  |  |  | 12 days |  |  | 0.149 | 0.082 | SD(Est), N | ^o^ |  |
|  |  |  | 14 days |  |  | 0.192 | 0.082 | SD(Est), N | ^o^ |  |
|  |  |  | 18 days |  |  | 0.220 | 0.082 | SD(Est), N | ^o^ |  |
|  |  |  | 22 days |  |  | 0.227 | 0.082 | SD(Est), N | ^o^ |  |
|  |  |  | 26 days |  |  | 0.234 | 0.082 | SD(Est), N | ^o^ |  |
|  |  |  | 30 days |  |  | 0.227 | 0.082 | SD(Est), N | 1.4 |  |
| MOFFA3 | None | M | 5 |  | 10 | 0.023 | 0.082 | SD(Est), N | 0.5 |  |
|  |  |  | 11 |  |  | 0.171 | 0.082 | SD(Est), N | -0.4 |  |
|  |  | F | 5 |  | 17 | -0.005 | 0.063 | SD(Est), N | 0.4 |  |
|  |  |  | 11 |  |  | 0.290 | 0.063 | SD(Est), N | 2.1 |  |
| NIAURA | None | F-E | 12 | 9 | 9 | 0.248 | 0.090 | CI |  | 1.1 |
|  |  | F-C |  |  | 9 | 0.129 | 0.082 | CI |  | 0.6 |
| NILSSO | None | B | 9 |  | 98 | 0.100 | 0.026 | SD(Est), N | 1.7 |  |
|  |  |  | 17 |  |  | 0.100 | 0.026 | SD(Est), N | 2.7 |  |
| NORREG | None | B | 6 ^[[16]](#endnote-16)^ | 4 | 75 | 0.300^[[17]](#endnote-17)^ | 0.030 | SD(Est), N | 1.1 |  |
| PRIEME | None | B-P | 4 |  | 29 | 0.130 | 0.038 | P Sig | 1.4 | 0.5 |
|  |  |  | 26 |  |  | 0.020 | 0.048 | SD(Est), N | 4.3 | 1.5 |
|  |  | B-R | 4 |  | 33 | 0.116 | 0.039 | SD, N | 1.4 | 0.5 |
| PULS | None | B | 5 | 2.5 | 144 | 0.000^j^ | 0.022 | SD(Est), N | 2.0 |  |
| QUENSE | None | M | 2 |  | 12 | 0.030 | 0.075 | SD(Est), N | 0.3 |  |
| RABKIN | None | M | 8-13 |  | 14 | 0.153 | 0.044 | Given | 2.0^[[18]](#endnote-18)^ |  |
|  |  | F |  |  | 21 | 0.080 | 0.057 | Given | 2.0^r^ |  |
| RAHILL | Age | M | 156 ^[[19]](#endnote-19)^ | 78 |  | 0.026^[[20]](#endnote-20)^ | 0.026 | P Sig |  |  |
| RICHAR | None | B | 3-12 av 5 |  | 58 | 0.130 | 0.034 | SD, N | 2.4 | 0.8 |
| SHENNA | None | M | 52-209 av 171 ^[[21]](#endnote-21)^ | 119 | 41 | 0.157^[[22]](#endnote-22)^ | 0.041 | SD(Est), N | 1.9 |  |
| STAMFO | None | F-P | 7 |  | 3 | 0.129 | 0.152 | SD(Est), N | 2.0 |  |
|  |  |  | 59 |  |  | 0.155 | 0.150 | SD(Est), N | 8.2 |  |
|  |  | F-R | 7 |  | 9 | 0.207 | 0.088 | SD(Est), N | 2.4 |  |
| STUBBE | None | M | 1 |  | 10 | 0.148^[[23]](#endnote-23)^ | 0.082 | SD(Est), N | ^o^ |  |
|  |  |  | 2 |  |  | 0.238^w^ | 0.082 | SD(Est), N | ^o^ |  |
|  |  |  | 4 |  |  | 0.238^w^ | 0.082 | SD(Est), N | ^o^ |  |
|  |  |  | 6 |  |  | 0.283^w,^ ^[[24]](#endnote-24)^ | 0.082 | SD(Est), N | 1.8 |  |
| SUWAZO | Age +3^[[25]](#endnote-25)^ | M | 52 | 26 | 445 | 0.044 | 0.012 | SD(Est), N | 1.6 | 0.5 |
|  |  |  | 104 | 78 |  | 0.065 | 0.012 | SD(Est), N | 1.9 | 0.7 |
|  |  |  | 156 | 130 |  | 0.072 | 0.012 | SD(Est), N | 1.9 | 0.6 |
| SWANK | None | F | 1 |  | 8 | 0.127 | 0.092 | SD(Est), N | 0.4 |  |
|  |  |  | 2 |  |  | 0.266 | 0.060 | Given | ^o^ |  |
|  |  |  | 3 |  |  | 0.243 | 0.055 | Given | -1.5 |  |
| TAMURA | Age | M | 209 | 26-52 | 26 | 0.160 | 0.051 | SD(Est), N | 2.4 | 0.9 |
|  |  |  | 209 | 53-104 | 46 | 0.106 | 0.038 | SD(Est), N | 2.1 | 0.7 |
|  |  |  | 209 | 105-209 | 45 | 0.060 | 0.039 | SD(Est), N | 2.5 | 0.9 |
| TERRES | None | B | 12 |  | 52 | ^[[26]](#endnote-26)^ |  |  |  |  |
|  |  |  | 23 |  |  | 0.054 | 0.034 | CI | 3.4 |  |
| TONSTA | None | B | 13 | 12 | 29 | 0.130 | 0.048 | SD(Est), N |  |  |
| VANDEN | None | M | 4 |  | 17 | 0.140 | 0.063 | SD(Est), N | ^o^ |  |
|  |  |  | 13 |  |  | 0.090 | 0.063 | SD(Est), N | 3.9 |  |
|  |  |  | 26 |  |  | 0.030 | 0.063 | SD(Est), N | ^o^ |  |
|  |  |  | 52 |  |  | 0.000 | 0.063 | SD(Est), N | 4.5 |  |
| YAMAMO | Age | M | 156 | 78 |  | ^[[27]](#endnote-27)^ |  |  |  |  |
| YEH | Sex, Age,  +6 ^[[28]](#endnote-28)^ | B | 156 | 78 | 380 | -0.020 | 0.020 | CI | 3.8 |  |
| YOON | None | M-L | 52-156 av 88 ^[[29]](#endnote-29)^ | 44 | 111 | 0.051 | 0.025 | SD(Est), N | ^[[30]](#endnote-30)^ |  |
|  |  | M-H |  |  | 115 | 0.039 | 0.025 | SD(Est), N | ^dd^ |  |
| ZHANG | None | M | 13-26 |  | 41 | 0.254^[[31]](#endnote-31)^ | 0.041 | SD(Est), N |  |  |
|  |  |  |  |  |  |  |  |  |  |  |

Data in the body of the table constitute the main data set analysed, except where only a footnote is shown for HDL-C change. All additional HDL-C change data available from the source papers are given in footnotes.

## Table A2-2 : Changes in HDL-C (mmol/l), bodyweight (kg) and BMI (kg/m^2^) in continuing smokers

| REF | Sex^[[32]](#endnote-32)^ | Period^[[33]](#endnote-33)^ | HDL-C | | | Weight change^[[34]](#endnote-34)^ | BMI change^c^ |
| --- | --- | --- | --- | --- | --- | --- | --- |
|  |  |  | N^[[35]](#endnote-35)^ | Change^[[36]](#endnote-36)^ | SE^[[37]](#endnote-37)^ |  |  |
|  |  |  |  |  |  |  |  |
| BASLER | B | 12 | 53 | -0.072 | 0.036 | 0.0 |  |
| BURNET | F | av 243^[[38]](#endnote-38)^ | 91 | 0.023 | 0.026 | 2.5 |  |
|  |  | av 300^[[39]](#endnote-39)^ | 84 | -0.013 | 0.023 | 3.2 |  |
| GERACE | M | 313 | 2060 | -0.008 | 0.006 | -0.6 |  |
| GREEN | M | 52-209 av 130 | 284 | 0.034^[[40]](#endnote-40)^ | 0.017 |  | 0.2 |
| HAUSTE | M | 4 | 33 | -0.024 | 0.045 |  |  |
|  |  | 8 |  | 0.000 | 0.045 |  |  |
|  |  | 12 |  | 0.037 | 0.045 |  |  |
|  |  | 26 |  | 0.056 | 0.045 |  |  |
| IINO | B | 52 | 16 | ^[[41]](#endnote-41)^ |  | 0.1 |  |
| KUSHIM | M | 261 | 378 | -0.204^[[42]](#endnote-42)^ | 0.016 |  | -0.1 |
| MOFFA1 | F | 4 | 8 | 0.000 | 0.092 | ^[[43]](#endnote-43)^ |  |
|  |  | 9 |  | -0.049 | 0.092 | ^l^ |  |
| MOFFA2 | M | 2 days | 6 | -0.014 | 0.106 | ^l^ |  |
|  |  | 4 days |  | -0.028 | 0.106 | ^l^ |  |
|  |  | 6 days |  | -0.057 | 0.106 | ^l^ |  |
|  |  | 8 days |  | 0.036 | 0.106 | ^l^ |  |
|  |  | 10 days |  | 0.057 | 0.106 | ^l^ |  |
|  |  | 12 days |  | 0.036 | 0.106 | ^l^ |  |
|  |  | 14 days |  | 0.043 | 0.106 | ^l^ |  |
|  |  | 18 days |  | 0.050 | 0.106 | ^l^ |  |
|  |  | 22 days |  | -0.014 | 0.106 | ^l^ |  |
|  |  | 26 days |  | -0.014 | 0.106 | ^l^ |  |
|  |  | 30 days |  | 0.043 | 0.106 | ^l^ |  |
|  | F | 2 days | 5 | 0.057 | 0.117 | ^l^ |  |
|  |  | 4 days |  | -0.043 | 0.117 | ^l^ |  |
|  |  | 6 days |  | -0.050 | 0.117 | ^l^ |  |
|  |  | 8 days |  | 0.000 | 0.117 | ^l^ |  |
|  |  | 10 days |  | -0.057 | 0.117 | ^l^ |  |
|  |  | 12 days |  | 0.007 | 0.117 | ^l^ |  |
|  |  | 14 days |  | -0.021 | 0.117 | ^l^ |  |
|  |  | 18 days |  | -0.028 | 0.117 | ^l^ |  |
|  |  | 22 days |  | -0.050 | 0.117 | ^l^ |  |
|  |  | 26 days |  | -0.035 | 0.117 | ^l^ |  |
|  |  | 30 days |  | 0.036 | 0.117 | ^l^ |  |
| NILSSO | B | 17 | 156 | 0.100 | 0.021 | 0.0 |  |
| PRIEME | B | 4 | 72 | 0.000 | 0.031 | 0.2 | 0.0 |
| RABKIN | M | 8-13 | 13 | 0.010 | 0.060 | 0.2^[[44]](#endnote-44)^ |  |
|  | F |  | 20 | 0.044 | 0.078 | 0.2^m^ |  |
| SUWAZO | M | 52 | 2403 | 0.008^[[45]](#endnote-45)^ | 0.005 | 0.2 | 0.1 |
|  |  | 104 |  | 0.010^n^ | 0.005 | 0.3 | 0.1 |
|  |  | 156 |  | 0.016^n^ | 0.005 | 0.5 | 0.2 |
| SWANK | F | 1 | 9 | -0.054 | 0.087 | 0.6 |  |
|  |  | 3 |  | -0.044 | 0.087 | 0.1 |  |
| TAMURA | M | 209 | 985 | 0.005 | 0.008 | 0.5 | 0.2 |
| VANDEN | M | 52 | 31 | 0.030 | 0.047 |  |  |
| YEH | B | 156 | 2018 | -0.070 | 0.005 | 0.6 |  |
| YOON | M | 52-156 av 83 | 950 | 0.030^[[46]](#endnote-46)^ | 0.008 | 0.2 | 0.1 |

## Table A2-3 : Changes in HDL-C (mmol/l), bodyweight (kg) and BMI (kg/m^2^) in never smokers

| REF | Sex^[[47]](#endnote-47)^ | Period^[[48]](#endnote-48)^ | HDL-C | | | Weight change^[[49]](#endnote-49)^ | BMI change^c^ |
| --- | --- | --- | --- | --- | --- | --- | --- |
|  |  |  | N^[[50]](#endnote-50)^ | Change^[[51]](#endnote-51)^ | SE^[[52]](#endnote-52)^ |  |  |
|  |  |  |  |  |  |  |  |
| BURNET | F | av 243 | 286 | 0.026 | 0.016 | 2.4 |  |
|  |  | av 300 | 258 | 0.029 | 0.016 | 3.5 |  |
| KUSHIM | M | 261 | 87 | -0.163^[[53]](#endnote-53)^ | 0.026 |  | 0.2 |
| MOFFA1 | F | 4 | 10 | 0.056 | 0.082 | ^[[54]](#endnote-54)^ |  |
|  |  | 9 |  | 0.035 | 0.082 | ^h^ |  |
| MOFFA2 | M | 2 days | 7 | 0.021 | 0.099 | ^h^ |  |
|  |  | 4 days |  | 0.007 | 0.099 | ^h^ |  |
|  |  | 6 days |  | 0.007 | 0.099 | ^h^ |  |
|  |  | 8 days |  | 0.05 | 0.099 | ^h^ |  |
|  |  | 10 days |  | 0.036 | 0.099 | ^h^ |  |
|  |  | 12 days |  | 0.036 | 0.099 | ^h^ |  |
|  |  | 14 days |  | 0.036 | 0.099 | ^h^ |  |
|  |  | 18 days |  | 0.007 | 0.099 | ^h^ |  |
|  |  | 22 days |  | -0.007 | 0.099 | ^h^ |  |
|  |  | 26 days |  | 0.057 | 0.099 | ^h^ |  |
|  |  | 30 days |  | 0.028 | 0.099 | ^h^ |  |
|  | F | 2 days | 5 | -0.007 | 0.117 | ^h^ |  |
|  |  | 4 days |  | 0.000 | 0.117 | ^h^ |  |
|  |  | 6 days |  | -0.021 | 0.117 | ^h^ |  |
|  |  | 8 days |  | 0.007 | 0.117 | ^h^ |  |
|  |  | 10 days |  | 0.000 | 0.117 | ^h^ |  |
|  |  | 12 days |  | 0.007 | 0.117 | ^h^ |  |
|  |  | 14 days |  | -0.035 | 0.117 | ^h^ |  |
|  |  | 18 days |  | -0.014 | 0.117 | ^h^ |  |
|  |  | 22 days |  | 0.007 | 0.117 | ^h^ |  |
|  |  | 26 days |  | -0.007 | 0.117 | ^h^ |  |
|  |  | 30 days |  | 0.028 | 0.117 | ^h^ |  |
| MOFFA3 | M | 5 | 7 | 0.013 | 0.099 | -0.7 |  |
|  |  | 11 |  | 0.013 | 0.099 | -0.4 |  |
|  | F | 5 | 9 | 0.044 | 0.087 | 0.1 |  |
|  |  | 11 |  | 0.026 | 0.087 | 0.5 |  |
| RAHILL | M | 156^[[55]](#endnote-55)^ | ^j^ | -0.002^[[56]](#endnote-56)^ | 0.015 |  |  |
| VANDEN | M | 52 | 25 | 0.090 | 0.052 |  |  |
| YEH | B | 156 | 4090 | -0.060 | 0.005 | 1.1 |  |

1. B = both sexes, F = females, M = males; L=low weight gain (<1.3 kg), H=high weight gain (≥1.3 kg); P = persistent quitter, R = resumed smoking before end of study; E = exercise training intervention, C = control group [↑](#endnote-ref-1)
2. Period between first measurement when smoking and second measurement when quitting. In weeks, except where indicated. “av” = average [↑](#endnote-ref-2)
3. Best estimate of time since quit at second measurement. Only shown if different from intra-period measurement. In weeks, except where indicated. “av” = average [↑](#endnote-ref-3)
4. N = number of quitters HDL-C estimate is based on. Where blank, the estimate refers to the same group of participants as the row above. Where different from earlier rows for the same study/sex, the estimate refers to a smaller group who continued to participate in the study for longer (BURNET, ELIAS1), or the participants were stratified by time since quit (TAMURA), by smoking resumption before the end of the study (MOFFA1, PRIEME, STAMFO), by intervention group (NIAURA), or by weight gain during the study (YOON). Changes in weight and BMI are based on essentially the same group of quitters, although there may have been a small difference in N due to missing data [↑](#endnote-ref-4)
5. Blank cell indicates data not available [↑](#endnote-ref-5)
6. SE Der = derivation of standard error as indicated by CI = from confidence interval; SD(Est), N = from estimated standard derivation and number of subjects; P Sig = from significance p-value; SD, N = from standard derivation and number of subjects [↑](#endnote-ref-6)
7. Additional estimates by intervention are : placebo patch 0.062 (SE 0.020, N=78), 7mg patch 0.028 (0.034, 60), 14 mg patch 0.057 (0.023, 134), 21 mg patch (0.079 (0.021, 160) ALLEN [↑](#endnote-ref-7)
8. Time to menopause + 1 year. Average based on quitters and continuing smokers combined BURNET [↑](#endnote-ref-8)
9. Time to menopause + 2 years. Average based on quitters and continuing smokers combined BURNET [↑](#endnote-ref-9)
10. Estimate is change in median FEHER, PULS [↑](#endnote-ref-10)
11. Stated not to have changed significantly FEHER [↑](#endnote-ref-11)
12. The change was stated to be significant in both men and women, no further details given. The change was 0.160 (SE=0.063, N=31) when restricted to those who did not change their exercise level FORTMA [↑](#endnote-ref-12)
13. Estimate is change relative to change in never smokers GREEN [↑](#endnote-ref-13)
14. HDL-C level stated not to have changed during the study IINO, KUME [↑](#endnote-ref-14)
15. For analysis, value was estimated by interpolation – see Methods [↑](#endnote-ref-15)
16. Smoking was allowed to the end of week 1 and some quitters “with slips” may have quit for only 2 weeks NORREG [↑](#endnote-ref-16)
17. Estimate is median change NORREG [↑](#endnote-ref-17)
18. Estimate is for sexes combined RABKIN [↑](#endnote-ref-18)
19. Results are adjusted to 3 year interval RAHILL [↑](#endnote-ref-19)
20. Estimate (and additional estimates) are relative to continuing smokers. Numbers of subjects and numbers of observations (see Table 2 of main paper) relevant to each estimate unknown, so SEs are approximate estimates. In a subgroup of 1386 men not taking cholesterol-modifying therapy, quitting smoking was stated to be associated with HDL-C increases. Additional estimates were 0.070 (0.030) for ages 32-67 and 0.029 (0.040) for ages 68+, adjusted for baseline HDL-C, LDL-C, diabetes, hypertension, CVD, CHD and medications RAHILL [↑](#endnote-ref-20)
21. Average time since quit was reported; time between first examination and quitting unknown, arbitrarily assumed 1 year SHENNA [↑](#endnote-ref-21)
22. Additional estimates stratified by time since quitting were 0.176 (N=22) for those quit 1-2 years, and a significant increase (N=19, p<0.01) for those quit >2 to 4 years SHENNA [↑](#endnote-ref-22)
23. Estimate based on %change STUBBE [↑](#endnote-ref-23)
24. Results were also presented showing for each person the maximum value and the week when it was attained (mean maximum HDL-C 0.370 (SE 0.059) at mean week 5.2) STUBBE [↑](#endnote-ref-24)
25. Adjusted for day/shift work, regular exercise and daily alcohol consumption SUWAZO [↑](#endnote-ref-25)
26. Described as “transient mean increase of 5 to 6 mg/dl” (0.129 to 0.155 mmol/l). Also shown in a graph which is too small to be read reliably but suggests the increase is significant TERRES [↑](#endnote-ref-26)
27. No estimate of HDL-C change available, but 49 (13.1%) out of the 373 men who quit smoking normalized their HDL-C (from below 40mg/dl to above this value), while 238 (7.9%) of 2998 who continued to smoke had a normalized HDL-C, giving an age-adjusted odds ratio 2.55 (95%CI 1.68-3.86) YAMAMO [↑](#endnote-ref-27)
28. Adjusted for race, location, education, physical activity, baseline HDL-C, and time between baseline and 3 year follow-up YEH [↑](#endnote-ref-28)
29. Information on period refers to whole study (not stratified by weight gain) YOON [↑](#endnote-ref-29)
30. Stratified on weight gain, so assumed in analysis to be in first and third tertiles respectively YOON [↑](#endnote-ref-30)
31. Additional estimates stratified by presence of comorbidities (hyperlipemia, hypertension, hyperglycemia) were 0.300 (N=22) without comorbidities and 0.200 (N=19) with comorbidities ZHANG [↑](#endnote-ref-31)
32. B = both sexes, F = females, M = males [↑](#endnote-ref-32)
33. Period between the two measurements in weeks, except where indicated. “av” = average [↑](#endnote-ref-33)
34. Blank cell indicates data not available [↑](#endnote-ref-34)
35. N = number of smokers HDL-C change estimate based on. Changes in weight and BMI are based on essentially the same group ofsmokers, although there may have been a small difference in N due to missing data. [↑](#endnote-ref-35)
36. mmol/l. Data are unadjusted unless otherwise stated [↑](#endnote-ref-36)
37. As given, or estimated by various methods [↑](#endnote-ref-37)
38. Time to menopause + 1 year. Average based on quitters and continuing smokers combined BURNET [↑](#endnote-ref-38)
39. Time to menopause + 2 years. Average based on quitters and continuing smokers combined BURNET [↑](#endnote-ref-39)
40. Estimate is change relative to never smokers, and is age adjusted. An additional estimate is 0.031 (SE 0.017) adjusted for age, BMI, alcohol and coffee consumption GREEN [↑](#endnote-ref-40)
41. No change reported (at p < 0.05) IINO [↑](#endnote-ref-41)
42. Adjustment unknown. An estimate additionally adjusted for change in BMI is -0.204 (SE 0.016) KUSHIM [↑](#endnote-ref-42)
43. Weight stated not to have changed during the study in continuing smokers MOFFA1, MOFFA2 [↑](#endnote-ref-43)
44. Estimate is for sexes combined RABKIN [↑](#endnote-ref-44)
45. Estimates are adjusted for day/shift work, regular exercise and daily alcohol consumption. Alternative estimates additionally adjusted for change in BMI from baseline are 0.010, 0.016 and 0.023 for 1, 2 and 3 year changes respectively SUWAZO [↑](#endnote-ref-45)
46. An alternative estimate adjusted for age, marital status, education, economic status, diabetes mellitus, hypertension, amount smoked, drinking behaviour, physical inactivity, and time interval between baseline and follow-up is 0.031 YOON [↑](#endnote-ref-46)
47. B = both sexes, F = females, M = males [↑](#endnote-ref-47)
48. Period between the two measurements in weeks, except where indicated. “av” = average [↑](#endnote-ref-48)
49. Blank cell indicates data not available [↑](#endnote-ref-49)
50. N = number of non-smokers HDL-C estimate based on. Changes in weight and BMI are based on essentially the same group of non-smokers, although there may have been a small difference in N due to missing data. [↑](#endnote-ref-50)
51. Data are unadjusted unless otherwise stated [↑](#endnote-ref-51)
52. As given, or estimated by various methods [↑](#endnote-ref-52)
53. Adjustment unknown. An estimate additionally adjusted for change in BMI is –0.152 (SE 0.020) KUSHIM [↑](#endnote-ref-53)
54. Weight stated not to have changed during the study in non-smokers MOFFA1, MOFFA2 [↑](#endnote-ref-54)
55. Results are adjusted to 3 year interval RAHILL [↑](#endnote-ref-55)
56. Numbers of subjects and numbers of observations (see Table 2 of main paper) relevant to each estimate unknown, so SEs are approximate estimates. Estimate (and additional estimates) are relative to continuing smokers, and are age adjusted. Additional estimates adjusted for LDL-C, diabetes mellitus, hypertension, cerebrovascular disease, coronary artery disease and medications were 0.013 (0.015) overall, 0.008 (0.019) for ages 32-67 and 0.026 (0.027) for ages 68+ RAHILL [↑](#endnote-ref-56)
