# Supplementary material for: The effect of quitting smoking on HDL-cholesterol - a review based on within-subject changes
Source: Biomark Res. 2013 Sep 13;1:26. doi: 10.1186/2050-7771-1-26 (PMC4177613; doi:10.1186/2050-7771-1-26)
Supplement: Additional file 3 — Further analyses. This file presents results of analyses of HDL-C change in quitters additional to those shown in Table 3. It also shows results of equivalent analyses for continuing and never smokers. [file 2050-7771-1-26-S3.docx]

**The effect of quitting smoking on HDL-Cholesterol - A review based on within-subject changes**

# Barbara A Forey, John S Fry, Peter N Lee, Alison J Thornton and Katharine J Coombs

# Additional File 3 – Further analyses

## Table 3 (extension) : Estimates of change in total HDL-C (mmol/dl) following quitting^a^

| Factor | Level | N^b^ | Unweighted analysis | | Inverse-variance weighted analysis | |
| --- | --- | --- | --- | --- | --- | --- |
|  |  |  | Mean (95% CI) | p^c^ | Mean (95% CI) | p^c^ |
| **Univariate analyses** |  |  |  |  |  |  |
| Max Age^f^ | <50 years | 8 | 0.137 (0.060 to 0.214) | NS | 0.188 (0.090 to 0.286) | 0.02 |
|  | 50-70 years | 28 | 0.103 (0.068 to 0.138) |  | 0.048 (0.026 to 0.070) |  |
|  | >70 years | 58 | 0.105 (0.076 to 0.133) |  | 0.064 (0.043 to 0.085) |  |
| Sex | Male | 46 | 0.087 (0.057 to 0.116) | 0.05 | 0.047 (0.029 to 0.065) | 0.015 |
|  | Female | 30 | 0.149 (0.109 to 0.189) |  | 0.138 (0.075 to 0.201) |  |
|  | Combined | 18 | 0.100 (0.060 to 0.140) |  | 0.073 (0.045 to 0.100) |  |
| Continent | N America | 49 | 0.125 (0.092 to 0.157) | 0.09 | 0.042 (0.022 to 0.062) | 0.016 |
|  | Europe | 30 | 0.116 (0.083 to 0.150) |  | 0.107 (0.074 to 0.140) |  |
|  | Asia | 13 | 0.055 (0.008 to 0.102) |  | 0.060 (0.036 to 0.085) |  |
|  | Other | 2 | 0.065 (-0.056 to 0.186) |  | 0.057 (-0.149 to 0.262) |  |
| Timing^g^ | <1990 | 26 | 0.113 (0.076 to 0.150) | NS | 0.028 (0.005 to 0.052) | 0.004 |
|  | 1990-1999 | 58 | 0.108 (0.078 to 0.137) |  | 0.082 (0.061 to 0.102) |  |
|  | 2000+ | 10 | 0.085 (0.025 to 0.144) |  | 0.067 (0.031 to 0.103) |  |
| Study type | Observational | 16 | 0.066 (0.023 to 0.109) | NS | 0.054 (0.031 to 0.078) | 0.09 |
|  | RCT | 16 | 0.117 (0.074 to 0.160) |  | 0.089 (0.058 to 0.120) |  |
|  | Other | 62 | 0.120 (0.092 to 0.148) |  | 0.047 (0.023 to 0.071) |  |
| Product smoked | Any | 16 | 0.101 (0.056 to 0.145) | NS | 0.070 (0.043 to 0.097) | NS |
|  | Cigarettes | 67 | 0.106 (0.079 to 0.133) |  | 0.049 (0.028 to 0.069) |  |
|  | Cigarettes only | 11 | 0.117 (0.059 to 0.175) |  | 0.076 (0.038 to 0.114) |  |
| Validation of quitting | Yes | 48 | 0.101 (0.067 to 0.134) | NS | 0.057 (0.033 to 0.081) | NS |
|  | No | 46 | 0.111 (0.083 to 0.138) |  | 0.061 (0.041 to 0.081) |  |
| Constraint on diet/exercise | Stay same | 15 | 0.147 (0.095 to 0.199) | NS | 0.195 (0.104 to 0.286) | 0.004 |
|  | Improve | 9 | 0.128 (0.070 to 0.185) |  | 0.037 (0.011 to 0.064) |  |
|  | No constraint | 70 | 0.094 (0.069 to 0.118) |  | 0.064 (0.047 to 0.082) |  |
| Baseline HDL-C | 1st tertile | 34 | 0.121 (0.083 to 0.158) | 0.05 | 0.087 (0.056 to 0.119) | <0.001 |
|  | 2nd tertile | 25 | 0.136 (0.093 to 0.179) |  | 0.103 (0.058 to 0.148) |  |
|  | 3rd tertile | 28 | 0.099 (0.066 to 0.132) |  | 0.070 (0.049 to 0.091) |  |
|  | unknown | 7 | 0.030 (-0.034 to 0.094) |  | 0.019 (-0.005 to 0.042) |  |
| Baseline weight/BMI^h^ | 1st tertile | 19 | 0.146 (0.082 to 0.210) | NS | 0.194 (0.077 to 0.312) | 0.08 |
|  | 2nd tertile | 28 | 0.086 (0.052 to 0.119) |  | 0.061 (0.039 to 0.084) |  |
|  | 3rd tertile | 21 | 0.108 (0.056 to 0.159) |  | 0.078 (0.034 to 0.123) |  |
|  | unknown | 26 | 0.119 (0.082 to 0.155) |  | 0.046 (0.022 to 0.070) |  |
| Change in weight/BMI^h^ | 1st tertile | 5 | 0.165 (0.087 to 0.243) | NS | 0.152 (0.085 to 0.219) | 0.02 |
|  | 2nd tertile | 29 | 0.083 (0.041 to 0.126) |  | 0.058 (0.008 to 0.109) |  |
|  | 3rd tertile | 51 | 0.104 (0.079 to 0.129) |  | 0.052 (0.036 to 0.068) |  |
|  | unknown | 9 | 0.121 (0.058 to 0.184) |  | 0.109 (0.044 to 0.174) |  |
| Baseline LDL-C | 1st tertile | 17 | 0.081 (0.016 to 0.146) | NS | 0.064 (0.019 to 0.109) | NS |
|  | 2nd tertile | 16 | 0.136 (0.067 to 0.206) |  | 0.088 (0.022 to 0.155) |  |
|  | 3rd tertile | 17 | 0.096 (0.052 to 0.139) |  | 0.084 (0.042 to 0.127) |  |
|  | unknown | 44 | 0.111 (0.082 to 0.140) |  | 0.052 (0.034 to 0.070) |  |
| Baseline Triglycerides | 1st tertile | 26 | 0.151 (0.105 to 0.197) | NS | 0.110 (0.023 to 0.196) | NS |
|  | 2nd tertile | 13 | 0.114 (0.067 to 0.162) |  | 0.114 (0.049 to 0.180) |  |
|  | 3rd tertile | 24 | 0.079 (0.034 to 0.124) |  | 0.065 (0.034 to 0.096) |  |
|  | unknown | 31 | 0.096 (0.064 to 0.128) |  | 0.051 (0.033 to 0.069) |  |
| Baseline Systolic BP | 1st tertile | 8 | 0.076 (0.015 to 0.138) | NS | 0.038 (-0.021 to 0.096) | NS |
|  | 2nd tertile | 7 | 0.116 (0.049 to 0.182) |  | 0.078 (0.041 to 0.115) |  |
|  | 3rd tertile | 5 | 0.053 (-0.025 to 0.131) |  | 0.059 (0.029 to 0.089) |  |
|  | unknown | 74 | 0.116 (0.091 to 0.141) |  | 0.056 (0.035 to 0.078) |  |
| Baseline Diastolic BP | 1st tertile | 8 | 0.076 (0.015 to 0.138) | NS | 0.038 (-0.021 to 0.096) | NS |
|  | 2nd tertile | 7 | 0.116 (0.049 to 0.182) |  | 0.078 (0.041 to 0.115) |  |
|  | 3rd tertile | 5 | 0.053 (-0.025 to 0.131) |  | 0.059 (0.029 to 0.089) |  |
|  | unknown | 74 | 0.116 (0.091 to 0.141) |  | 0.056 (0.035 to 0.078) |  |

See Table 3 in main paper for footnotes

## Additional Table A : Estimates of change in total HDL-C (mmol/dl) in continuing smokers^a^

| Factor | Level | N^b^ | Unweighted analysis | | Inverse-variance weighted analysis | |
| --- | --- | --- | --- | --- | --- | --- |
|  |  |  | Mean (95% CI) | p^c^ | Mean (95% CI) | p^c^ |
| Overall |  | 47 | -0.003 (-0.023 to 0.016) | NS | -0.014 (-0.037 to 0.009) | NS |
| **Univariate models** |  |  |  |  |  |  |
| Period^d^ | <3 weeks | 17 | -0.002 (-0.048 to 0.044) | NS | 0.031 (-0.144 to 0.206) | NS |
|  | 3 to <6 | 10 | -0.012 (-0.050 to 0.027) |  | -0.003 (-0.164 to 0.158) |  |
|  | 6 to <13 | 6 | -0.005 (-0.047 to 0.037) |  | 0.009 (-0.146 to 0.163) |  |
|  | 13 to <27 | 2 | 0.078 (0.005 to 0.151) |  | 0.069 (-0.089 to 0.227) |  |
|  | 27 to <52 | 0 |  |  |  |  |
|  | 52+ weeks | 12 | -0.012 (-0.041 to 0.018) |  | -0.015 (-0.039 to 0.008) |  |
| Time quit^e^ | not relevant |  |  |  |  |  |
| Max Age^f^ | <50 years | 6 | -0.005 (-0.048 to 0.039) | NS | 0.005 (-0.320 to 0.329) | 0.07 |
|  | 50-70 years | 9 | -0.015 (-0.051 to 0.021) |  | -0.034 (-0.061 to -0.007) |  |
|  | >70 years | 32 | 0.002 (-0.026 to 0.030) |  | 0.014 (-0.018 to 0.046) |  |
| Sex | Male | 25 | 0.004 (-0.024 to 0.031) | NS | 0.002 (-0.021 to 0.025) | 0.04 |
|  | Female | 18 | -0.013 (-0.049 to 0.025) |  | 0.002 (-0.153 to 0.156) |  |
|  | Combined | 4 | -0.011 (-0.063 to 0.042) |  | -0.059 (-0.098 to -0.021) |  |
| Continent | N America | 32 | -0.007 (-0.035 to 0.020) | NS | -0.041 (-0.072 to -0.010) | 0.07 |
|  | Europe | 8 | 0.016 (-0.021 to 0.053) |  | 0.038 (-0.079 to 0.155) |  |
|  | Asia | 7 | -0.015 (-0.054 to 0.025) |  | 0.005 (-0.023 to 0.033) |  |
|  | Other | 0 |  |  |  |  |
| Timing^g^ | <1990 | 10 | -0.023 (-0.056 to 0.010) | NS | -0.045 (-0.072 to -0.019) | 0.012 |
|  | 1990-1999 | 33 | 0.002 (-0.024 to 0.028) |  | 0.014 (-0.010 to 0.039) |  |
|  | 2000+ | 4 | 0.017 (-0.035 to 0.069) |  | 0.018 (-0.299 to 0.335) |  |
| Study type | Observational | 10 | -0.016 (-0.050 to 0.017) | NS | -0.018 (-0.045 to 0.010) | NS |
|  | RCT | 5 | 0.016 (-0.031 to 0.064) |  | 0.040 (-0.103 to 0.183) |  |
|  | Other | 32 | -0.002 (-0.030 to 0.026) |  | -0.007 (-0.061 to 0.047) |  |
| Product smoked | Any | 4 | 0.010 (-0.043 to 0.063) | NS | 0.010 (-0.024 to 0.045) | NS |
|  | Cigarettes | 39 | -0.008 (-0.031 to 0.015) |  | -0.031 (-0.059 to -0.002) |  |
|  | Cigarettes only | 4 | 0.017 (-0.036 to 0.070) |  | 0.018 (-0.367 to 0.402) |  |
| Validation of smoking | Yes | 36 | -0.010 (-0.034 to 0.015) | NS | -0.018 (-0.044 to 0.009) | NS |
|  | No | 11 | 0.011 (-0.021 to 0.042) |  | 0.000 (-0.051 to 0.051) |  |
| Constraint on diet/exercise | Stay same | 2 | -0.049 (-0.122 to 0.024) | NS | -0.049 (-0.904 to 0.805) | NS |
|  | Improve | 3 | -0.025 (-0.085 to 0.035) |  | -0.005 (-0.051 to 0.041) |  |
|  | No constraint | 42 | 0.002 (-0.019 to 0.023) |  | -0.018 (-0.047 to 0.011) |  |
| Baseline HDL-C | 1st tertile | 18 | 0.023 (-0.009 to 0.054) | 0.07 | 0.054 (-0.061 to 0.168) | NS |
|  | 2nd tertile | 16 | -0.002 (-0.037 to 0.034) |  | 0.030 (-0.047 to 0.107) |  |
|  | 3rd tertile | 9 | -0.022 (-0.055 to 0.010) |  | -0.019 (-0.048 to 0.009) |  |
|  | unknown | 4 | -0.050 (-0.099 to -0.002) |  | -0.028 (-0.077 to 0.021) |  |
| Baseline weight/BMI^h^ | 1st tertile | 13 | -0.014 (-0.069 to 0.041) | NS | 0.002 (-0.731 to 0.735) | NS |
|  | 2nd tertile | 13 | -0.001 (-0.031 to 0.029) |  | -0.012 (-0.042 to 0.018) |  |
|  | 3rd tertile | 12 | 0.016 (-0.047 to 0.079) |  | 0.027 (-0.410 to 0.464) |  |
|  | unknown | 9 | -0.011 (-0.047 to 0.025) |  | -0.022 (-0.073 to 0.029) |  |
| Change in weight/BMI^h^ | 1st tertile | 29 | -0.011 (-0.042 to 0.021) | NS | -0.018 (-0.068 to 0.033) | NS |
|  | 2nd tertile | 11 | -0.004 (-0.037 to 0.029) |  | -0.014 (-0.044 to 0.016) |  |
|  | 3rd tertile | 2 | 0.005 (-0.071 to 0.081) |  | 0.000 (-0.231 to 0.230) |  |
|  | unknown | 5 | 0.020 (-0.028 to 0.068) |  | 0.023 (-0.307 to 0.353) |  |
| Baseline LDL-C | 1st tertile | 11 | -0.011 (-0.077 to 0.055) | NS | 0.045 (-1.042 to 1.132) | NS |
|  | 2nd tertile | 2 | 0.032 (-0.041 to 0.104) |  | 0.031 (-0.040 to 0.102) |  |
|  | 3rd tertile | 14 | 0.024 (-0.022 to 0.070) |  | 0.063 (-0.088 to 0.213) |  |
|  | unknown | 20 | -0.016 (-0.039 to 0.007) |  | -0.021 (-0.046 to 0.004) |  |
| Baseline Triglycerides | 1st tertile | 3 | -0.016 (-0.081 to 0.048) | NS | -0.003 (-0.267 to 0.262) | NS |
|  | 2nd tertile | 13 | 0.017 (-0.035 to 0.070) |  | 0.087 (-0.083 to 0.258) |  |
|  | 3rd tertile | 14 | 0.007 (-0.041 to 0.054) |  | -0.039 (-0.077 to -0.001) |  |
|  | unknown | 17 | -0.012 (-0.039 to 0.015) |  | -0.003 (-0.031 to 0.025) |  |
| Baseline Systolic BP | 1st tertile | 3 | 0.012 (-0.050 to 0.074) | NS | -0.044 (-0.079 to -0.008) | 0.08 |
|  | 2nd tertile | 3 | 0.021 (-0.041 to 0.083) |  | 0.029 (-0.032 to 0.090) |  |
|  | 3rd tertile | 5 | -0.002 (-0.050 to 0.046) |  | 0.010 (-0.025 to 0.046) |  |
|  | unknown | 36 | -0.009 (-0.034 to 0.017) |  | -0.026 (-0.069 to 0.016) |  |
| Baseline Diastolic BP | 1st tertile | 3 | 0.012 (-0.050 to 0.074) | NS | -0.044 (-0.079 to -0.008) | 0.08 |
|  | 2nd tertile | 3 | 0.021 (-0.041 to 0.083) |  | 0.029 (-0.032 to 0.090) |  |
|  | 3rd tertile | 5 | -0.002 (-0.050 to 0.046) |  | 0.010 (-0.025 to 0.046) |  |
|  | unknown | 36 | -0.009 (-0.034 to 0.017) |  | -0.026 (-0.069 to 0.016) |  |

See Table 3 in main paper for footnotes, except footnote a : see Table A2-2 for data used on change, and footnote c : for the overall analysis test for an increase between measurements

## Additional Table B : Estimates of change in total HDL-C (mmol/dl) in never (or non) smokers^a^

| Factor | Level | N^b^ | Unweighted analysis | | Inverse-variance weighted analysis | |
| --- | --- | --- | --- | --- | --- | --- |
|  |  |  | Mean (95% CI) | p^c^ | Mean (95% CI) | p^c^ |
| Overall |  | 34 | 0.008 (-0.027 to 0.043) | NS | -0.048 (-0.076 to -0.020) | 0.004 |
| **Univariate models** |  |  |  |  |  |  |
| Period^d^ | <3 weeks | 16 | 0.021 (-0.043 to 0.084) | NS | 0.033 (-0.250 to 0.316) | NS |
|  | 3 to <6 | 9 | 0.028 (-0.027 to 0.083) |  | 0.034 (-0.247 to 0.315) |  |
|  | 6 to <13 | 3 | 0.022 (-0.053 to 0.096) |  | 0.019 (-0.266 to 0.303) |  |
|  | 13 to <27 | 0 |  |  |  |  |
|  | 27 to <52 | 0 |  |  |  |  |
|  | 52+ weeks | 6 | -0.013 (-0.066 to 0.039) |  | -0.049 (-0.078 to -0.019) |  |
| Time quit^e^ | not relevant |  |  |  |  |  |
| Max Age^f^ | <50 years | 0 |  | 0.03 |  | NS |
|  | 50-70 years | 4 | -0.042 (-0.095 to 0.011) |  | -0.054 (-0.082 to -0.026) |  |
|  | >70 years | 30 | 0.028 (-0.006 to 0.061) |  | 0.007 (-0.073 to 0.088) |  |
| Sex | Male | 16 | -0.003 (-0.056 to 0.051) | NS | -0.033 (-0.096 to 0.030) | 0.07 |
|  | Female | 17 | 0.028 (-0.023 to 0.080) |  | 0.028 (-0.045 to 0.101) |  |
|  | Combined | 1 | -0.060 (-0.187 to 0.067) |  | -0.060 (-0.086 to -0.034) |  |
| Continent | N America | 32 | 0.015 (0.001 to 0.030) | <0.001 | -0.045 (-0.072 to -0.018) | NS |
|  | Europe | 1 | 0.090 (0.033 to 0.147) |  | 0.090 (-0.217 to 0.397) |  |
|  | Asia | 1 | -0.163 (-0.220 to -0.106) |  | -0.163 (-0.315 to -0.011) |  |
|  | Other | 0 |  |  |  |  |
| Timing^g^ | <1990 | 7 | -0.016 (-0.064 to 0.033) | NS | -0.049 (-0.078 to -0.021) | NS |
|  | 1990-1999 | 27 | 0.029 (-0.018 to 0.077) |  | 0.054 (-0.175 to 0.283) |  |
|  | 2000+ | 0 |  |  |  |  |
| Study type | Observational | 5 | -0.034 (-0.082 to 0.014) | 0.03 | -0.050 (-0.078 to -0.021) | NS |
|  | RCT | 0 |  |  |  |  |
|  | Other | 29 | 0.031 (-0.005 to 0.066) |  | 0.053 (-0.155 to 0.260) |  |
| Product smoked | not relevant |  |  |  |  |  |
| Validation of quitting/ smoking | not relevant |  |  |  |  |  |
| Constraint on diet/exercise | Stay same | 0 |  |  |  |  |
|  | Improve | 0 |  |  |  |  |
|  | No constraint | 34 |  |  |  |  |
| Baseline HDL-C | 1st tertile | 0 |  | NS |  | NS |
|  | 2nd tertile | 14 | 0.036 (-0.030 to 0.102) |  | 0.064 (-0.181 to 0.309) |  |
|  | 3rd tertile | 16 | 0.013 (-0.043 to 0.070) |  | -0.059 (-0.089 to -0.030) |  |
|  | unknown | 4 | -0.028 (-0.092 to 0.037) |  | -0.011 (-0.069 to 0.046) |  |
| Baseline weight/BMI^h^ | 1st tertile | 13 | 0.026 (-0.067 to 0.118) | NS | 0.034 (-0.430 to 0.498) | NS |
|  | 2nd tertile | 4 | 0.024 (-0.063 to 0.110) |  | 0.025 (-0.424 to 0.474) |  |
|  | 3rd tertile | 13 | 0.019 (-0.062 to 0.100) |  | -0.058 (-0.093 to -0.024) |  |
|  | unknown | 4 | -0.028 (-0.100 to 0.044) |  | -0.012 (-0.080 to 0.056) |  |
| Change in weight/BMI^h^ | 1st tertile | 26 | 0.022 (-0.033 to 0.077) | NS | 0.027 (-0.172 to 0.225) | 0.03 |
|  | 2nd tertile | 4 | -0.045 (-0.113 to 0.023) |  | -0.064 (-0.084 to -0.043) |  |
|  | 3rd tertile | 2 | 0.027 (-0.063 to 0.117) |  | 0.027 (-0.031 to 0.086) |  |
|  | unknown | 2 | 0.044 (-0.046 to 0.134) |  | 0.005 (-0.054 to 0.064) |  |
| Baseline LDL-C | 1st tertile | 11 | 0.006 (-0.125 to 0.137) | NS | 0.011 (-0.890 to 0.911) | NS |
|  | 2nd tertile | 11 | 0.025 (-0.106 to 0.156) |  | 0.025 (-0.737 to 0.786) |  |
|  | 3rd tertile | 1 | 0.090 (-0.053 to 0.233) |  | 0.090 (-0.314 to 0.494) |  |
|  | unknown | 11 | -0.003 (-0.051 to 0.045) |  | -0.049 (-0.084 to -0.015) |  |
| Baseline Triglycerides | 1st tertile | 14 | 0.042 (-0.029 to 0.114) | NS | 0.069 (-0.167 to 0.304) | NS |
|  | 2nd tertile | 12 | -0.010 (-0.100 to 0.080) |  | -0.060 (-0.089 to -0.031) |  |
|  | 3rd tertile | 0 |  |  |  |  |
|  | unknown | 8 | -0.005 (-0.056 to 0.047) |  | -0.010 (-0.065 to 0.045) |  |
| Baseline Systolic BP | 1st tertile | 0 |  | NS |  | NS |
|  | 2nd tertile | 1 | -0.060 (-0.179 to 0.059) |  | -0.060 (-0.089 to -0.031) |  |
|  | 3rd tertile | 1 | 0.090 (-0.029 to 0.209) |  | 0.090 (-0.206 to 0.386) |  |
|  | unknown | 32 | 0.008 (-0.028 to 0.043) |  | -0.009 (-0.063 to 0.045) |  |
| Baseline Diastolic BP | 1st tertile | 0 |  | NS |  | NS |
|  | 2nd tertile | 1 | -0.060 (-0.179 to 0.059) |  | -0.060 (-0.089 to -0.031) |  |
|  | 3rd tertile | 1 | 0.090 (-0.029 to 0.209) |  | 0.090 (-0.206 to 0.386) |  |
|  | unknown | 32 | 0.008 (-0.028 to 0.043) |  | -0.009 (-0.063 to 0.045) |  |

See Table 3 in main paper for footnotes, except footnote a : see Table A2-2 for data used on change, and footnote c : for the overall analysis test for an increase between measurements.
